# Single-cell profiling of GP2-enriched pancreatic progenitors to simultaneously create acinar, ductal, and endocrine organoids

**DOI:** 10.7150/thno.78323

**Published:** 2023-03-21

**Authors:** Sarah Merz, Markus Breunig, Michael Karl Melzer, Sandra Heller, Sandra Wiedenmann, Thomas Seufferlein, Matthias Meier, Jana Krüger, Medhanie A Mulaw, Meike Hohwieler, Alexander Kleger

**Affiliations:** 1 Institute of Molecular Oncology and Stem Cell Biology, Ulm University Hospital, Ulm, Germany; 2 Department of Urology, Ulm University Hospital, Ulm, Germany; 3 Helmholtz Pioneer Campus, Helmholtz Zentrum München, Neuherberg, Germany; 4 Department of Internal Medicine I, Ulm University Hospital, Ulm, Germany; 5 Central Unit Single Cell Sequencing, Medical Faculty, Ulm University, Ulm, Germany; 6 Division of Interdisciplinary Pancreatology, Department of Internal Medicine I, Ulm University Hospital, Ulm, Germany; 7 Core Facility Organoids, Ulm University, Ulm, Germany

**Keywords:** Multipotent pancreatic progenitors, pancreatic organoids, *in vitro* differentiation, GP2, pancreatic acinar-like organoids

## Abstract

**Rationale:** Pancreatic lineage specification follows the formation of tripotent pancreatic progenitors (PPs). Current protocols rebuilding PPs *in vitro* have an endocrine lineage bias and are mostly based on PDX1/NKX6-1 coexpression neglecting other markers decisive for PP heterogeneity and lineage potential. However, true tripotent PPs are of utmost interest to study also exocrine disorders such as pancreatic cancer and to simultaneously generate all three pancreatic lineages from the same ancestor.

**Methods:** Here, we performed a comprehensive compound testing to advance the generation of multipotent progenitors, which were further characterized for their trilineage potential *in vitro* and* in vivo*. The heterogeneity and cell-cell communication across the PP subpopulations were analyzed via single-cell transcriptomics.

**Results:** We introduce a novel PP differentiation platform based on a comprehensive compound screening with an advanced *design of experiments* computing tool to reduce impurities and to increase Glycoprotein-2 expression and subsequent trilineage potential. Superior PP tripotency was proven *in vitro* by the generation of acinar, endocrine, and ductal cells as well as *in vivo* upon orthotopic transplantation revealing all three lineages at fetal maturation level. GP2 expression levels at PP stage ascribed varying pancreatic lineage potential. Intermediate and high GP2 levels were superior in generating endocrine and duct-like organoids (PDLO). FACS-based purification of the GP2^high^ PPs allowed the generation of pancreatic acinar-like organoids (PALO) with proper morphology and expression of digestive enzymes. scRNA-seq confirmed multipotent identity, positioned the GP2/PDX1/NKX6-1^high^ population next to human fetal tip and trunk progenitors and identified novel ligand-receptor (LR) interactions in distinct PP subpopulations. LR validation experiments licensed midkine and VEGF signaling to increase markers labelling the single cell clusters with high GP2 expression.

**Conclusion:** In this study, we guide human pluripotent stem cells into multipotent pancreatic progenitors. This common precursor population, which has the ability to mature into acinar, ductal and functional β-cells, serves as a basis for studying developmental processes and deciphering early cancer formation in a cell type-specific context. Using single-cell RNA sequencing and subsequent validation studies, we were able to dissect PP heterogeneity and specific cell-cell communication signals.

## Introduction

The early developmental events of human pancreatogenesis are poorly understood due to scarce tissue and data availability for ethical reasons. Consequently, most knowledge is gained from rodent data, although relevant differences exist across various species 1. During early pancreas development, multipotent pancreatic progenitor (PP) cells serve as a common ancestor in the pancreatic bud, further specifying into a trunk and a tip domain (2; reviewed in 3). Subsequently, the tip domain matures into acinar cells, whereas the endocrine and ductal cells originate from the bipotent trunk domain 3. The opportunity to culture human embryonic stem cells (hESC) from human preimplantation embryos 4 and to reprogram any human somatic cell to induced pluripotent stem cells (iPSCs) 5, 6 has paved the way for several multi-step pancreatic differentiation protocols, which allow the generation of high PP yields 7-10. The key advent across all these studies was the identification of the inductive cues leading to the upregulation of NKX6-1 in the PDX1-positive pancreatic endoderm (PE) 11, 12. Both transcription factors are also expressed in the pancreatic bud 13, harboring tri-potent pancreatic progenitors. The *in vitro* differentiated counterparts indeed enabled for the first time the production of functional endocrine cells including β- 9, 11, 14-19 and α-cells 20 for diabetes research and treatment. While only few papers reported immature and undirected exocrine differentiation 21, 22, others and we have most recently succeeded in generating virtually pure ductal cells 23-26. In contrast, acinar differentiation studies are limited in number and expression of maturation markers 21, 25, 27. In line, orthotopic transplantation of PPs into murine hosts yielded only few immature acinar cells indicating a lack of true multipotency within the engrafted PPs 21, 28, 29. Intriguingly, NKX6-1/PDX1-double-positive PPs can be produced with similarly high efficiencies (i) by developmentally opposing signaling cues (e.g. WNT or MEK inhibition vs. activation), (ii) with contrary timing across the developmental intermediates and (iii) in varying culture formats (2D vs. 3D) 7-9, 11. Thus, the entire field mainly relies on a dual protein marker combination, thereby neglecting the heterogeneity in pancreatic progenitor cells. However, two markers cannot be sufficient to engineer a cell population with trilineage potential giving rise to a cellular ecosystem as complex as the human pancreas. Supporting this assumption, heterogeneity within the definitive endoderm (DE) restricts subsequent endocrine lineage potential 16 and even segregates opposing lineages (e.g. pancreas vs. liver) 30. Moreover, even the expression level of NKX6-1 *per se* determines subsequent lineage potential 12, 31. Most recently, a preprinted study performed large-scale single cell transcriptomics for a number of human pluripotent stem cell (hPSC)-derived PPs and found pancreatic cell types at varying levels of maturity due to asynchronous differentiation accounting for a large fraction of the PP heterogeneity 32. Thus, well-defined and trilineage competent PPs are critical to truthfully mimic human fetal pancreatogenesis and to study pancreatic diseases, such as diabetes or pancreatic cancer. Particularly studying pancreatic carcinogenesis using proportionally induced ductal and acinar cells arising from the same truly multipotent PP ancestor would allow studying cancer cell origin relative to a given oncogenic hit. Interestingly, glycoprotein-2 (GP2) has been identified as a label for multipotent pancreatic progenitors 33. Current strategies utilizing GP2 for the enhancement of pancreatic *in vitro* differentiation, however, were based on cell sorting and evidence for improved exocrine lineage commitment is lacking 34, 35. Also, the signaling pathways determining GP2 expression levels in PPs and its molecular function are unknown.

Here, we employed the advanced *design of experiments* computing tool for stage-specific compound screening to develop a protocol that generates high yields of GP2-positive PPs with minimal lineage bias or contamination. In rigorous head-to-head comparisons, these progenitors proved their capacity for endocrine and exocrine lineage commitment *in vitro* and *in vivo*. Advanced ductal-like organoid maturation, glucose-responsive β-cells and pancreatic acinar-like organoids (PALOs) could be generated from the same PP ancestors. Single-cell transcriptomics of PPs did not only confirm their multipotent nature but also revealed the heterogeneity and complexity of early lineage determination. Close clustering to human fetal pancreas cells verifies our system for modeling human pancreas development and pancreatic diseases *in vitro*. Detailed ligand-receptor analysis and subsequent validation studies revealed unique, and so far unknown, cell-cell communication pathways within and between heterogeneous progenitor clusters, providing a comprehensive resource to identify and purify specific progenitor subtypes.

## Results

### Comprehensive compound testing fine-tunes lineage potential in pancreatic progenitors

**Figure 1:**
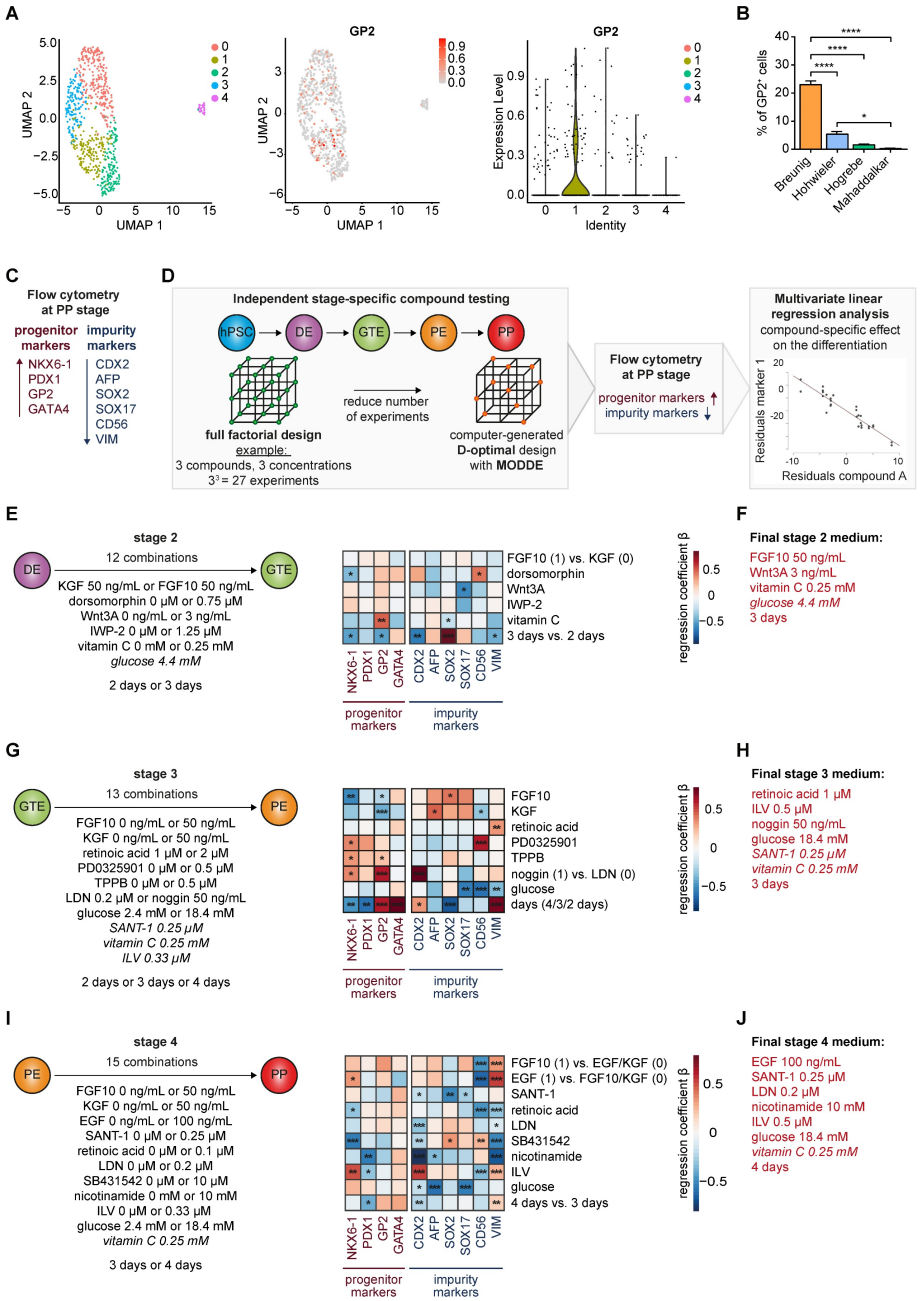
** Stage-specific compound testing for efficient generation of pancreatic progenitors derived from human pluripotent stem cells.** (**A**) UMAP plots of a published PP single-cell dataset and GP2 marker expression across the 5 different clusters, shown as UMAP and violin plot 36. (**B**) GP2 levels in PPs generated using existing protocols 14, 16, 21, 23, 24 were measured by flow cytometry. Mean ± SEM, n=3; p < 0.05: *, p < 0.0001: ****. (**C**) Extended flow cytometry marker panel for evaluation of PP generation by progenitor markers (NKX6-1, PDX1, GP2, GATA4) and impurity markers (CDX2, AFP, SOX2, SOX17, CD56, VIM). (**D**) Workflow of the compound testing in a D-optimal experimental setup generated with the software program MODDE (V12.1; Sartorius). Flow cytometry results for PP and impurity markers were further subjected to a multivariate linear regression analysis. (**E-J**) Stage-specific heatmaps illustrate the positive or negative impact of the specific compound on each marker. The heatmap is based on standardized regression coefficients (β) obtained by multiple linear regression analysis. (**E-F**) Multifactorial compound screen of stage 2 was performed with 12 different media compositions. For the direct comparison of FGF10 and KGF, FGF10 and KGF were encoded as 1 and 0, respectively. (**G-H**) Testing of stage 3 compounds was performed in 13 different approaches. Noggin (50 ng/mL) and LDN-193189 (0.2 µM) were encoded as 1 and 0, respectively. (**I-J**) Stage 4 media composition was tested in 15 combinations. Since either FGF10, EGF or KGF was used, 0 and 1 encoding was used for regression analysis. Compounds that are written *in italics* were not varied. All different media compositions were tested in duplicates of one differentiation. (**F, H, J**) Final stage 2, 3, and 4 media compositions are highlighted in red. p < 0.05: *, p < 0.01: **, p < 0.001: ***. DE: definitive endoderm; GP2: glycoprotein 2; GTE: gut tube endoderm; ILV: Indolactam V; LDN: LDN-193189; PE: pancreatic endoderm; PP: pancreatic progenitor; VIM: vimentin.

Multiple PP differentiation protocols employ a multitude of different growth factor combinations, concentrations, and timelines reaching similar NKX6-1/PDX1 double positive cell yields, the current mainstay in labelling pancreatic progenitors (**Figure S1A-E**) 14-16, 21, 23, 24. However, relevant cell heterogeneity in NKX6-1/PDX1 double positive PPs can be observed in scRNA-seq data leaving a potentially existing lineage bias largely unexplored (**Figure 1A**; **Figure S1F**) 36, 37. Recently, GP2 was introduced as a novel marker co-expressed in multipotent pancreatic progenitor cells. However, the number of GP2 positive cells was generally low across most tested protocols, with our own protocol standing out, still lower than 25% (**Figure 1A-B**) 23, 24, 36. To better define and modulate the arising PP populations, we used this previously published protocol 23, 24 as a starting point for stage-specific compound testing, followed by flow cytometry (FC)-based measurements of NKX6-1 and PDX1, together with the tip-progenitor marker GATA4 1 and the multipotency label GP2 at the pancreatic progenitor stage 33, 35. Alternate lineage fates were assessed by quantification of CDX2 (duodenal, intestine), AFP (liver), SOX2 (lung), SOX17 (residual endoderm), and CD56/vimentin (VIM) (mesoderm) (**Figure 1C**).

*Definitive endoderm and gut tube endoderm*: As changes in media composition at definitive endoderm stage did not relevantly alter GP2 yields (**Figure S2A-B, Table S1**), we started to directly approach the more complex stages 2, 3 and 4. A D-optimal design, calculated with the computing tool MODDE to strive the experimental setting for optimized conditions was applied. Thereby, the number of conditions could be restricted to a pre-defined set of growth factor combinations (**Figure 1D**). The flow cytometry results at the PP stage were processed using a multivariate linear regression analysis, allowing us to decipher the effect of each compound on expanding the GP2 population but suppressing the non-pancreatic lineages. Substitution of FGF10 by KGF during stage 2, did not significantly change PP and impurity marker composition (**Figure 1E, Table S2**). BMP (bone morphogenic protein) inhibition via dorsomorphin decreased NKX6-1 and increased the CD56-positive mesodermal cell population. Interestingly, both WNT activation (Wnt3A) and inhibition (IWP-2) resulted in similar expression profiles and limited the amount of SOX17-positive residual endoderm. Adding vitamin C had the strongest impact during stage 2 for promoting the multipotent GP2-positive population. The choice of the ideal duration of stage 2 was conflictive with short incubation reducing anteriorization of the foregut endoderm and longer incubation reducing intestinal and mesodermal marker expression. However, GP2 and NKX6-1 expression were reduced with prolonged stage 2 induction. Based on these results, we selected a final stage 2 medium containing FGF10 (50 ng/mL), Wnt3A (3 ng/mL), vitamin C (0.25 mM), and glucose (4.4 mM), applied for 3 days (**Figure 1F**).

*Pancreatic endoderm and pancreatic progenitor stage*: During stage 3, FGF10, KGF and an increased retinoic acid (RA) concentration impaired pancreatic endoderm formation, with decreased GP2 and NKX6-1 populations and increased impurity markers at the PP stage (**Figure 1G**, **Table S3**). We also decided against inhibiting MEK/ERK signaling via PD0325901 as increased PP marker expression was paralleled by contaminating CD56+ mesodermal cells. Protein kinase C (PKC) activation via TPPB or ILV substantially promoted GP2 and other PP marker expression (**Figure 1G, Figure S3A-B, Table S5**).

Furthermore, BMP inhibition by noggin treatment in stage 3 and LDN-193189 (LDN) during stage 4 tended to be most effective to increase GP2 and suppress CDX2 (**Figure 1G, I**). In addition, a high glucose environment was beneficial at both stage 3 and 4 to reduce non-pancreatic cell contaminations. A prolonged stage 3 was also tested with heterogeneous outcome: prolonged incubation caused a strong increase in the percentage of GP2 and GATA4 positive progenitors, whereas NKX6-1 and PDX1 decreased in parallel. Similar conflicting results were obtained for the non-pancreatic lineage marker expression. Based on the results of the compound testing and considering the partially opposing effects, we decided to use the following final stage 3 medium, applied for 3 days: retinoic acid (1 µM), ILV (0.5 µM), noggin (50 ng/mL), SANT-1 (0.25 µM), vitamin C (0.25 mM), and glucose (18.4 mM) (**Figure 1H**).

We finally investigated the transition from the pancreatic endoderm to pancreatic progenitors (**Figure 1I, Table S4**). In comparison to KGF, EGF and FGF10 revealed very similar expression profiles. While increasing PP markers, both growth factors were also strongly affecting mesodermal markers with a reduced CD56 but opposingly increased VIM expression. As EGF significantly increased the NKX6-1 positive cell number, we kept EGF in the final protocol. RA and hedgehog inhibition by SANT-1 suppressed alternate/non-pancreatic lineages, but RA also decreased NKX6-1 yield. TGF-β inhibition via SB431542 operated overall detrimentally. Vice versa, nicotinamide reduced the lineage bias. Longer stage 4 differentiation reduced CDX2 but also PDX1 positive cells, and induced VIM positive mesodermal cells. Given the impact of PKC activation on GP2 and NKX6-1 expression, we consecutively altered compounds and dosage to drive PKC activation during stage 3 and/or 4. TPPB was inferior to ILV and higher ILV concentrations were more effective to drive GP2 population expansion (**Figure S3A-B, Table S5**). Based on the results we decided for the following final stage 4 medium, applied for 4 days: EGF (100 ng/mL), SANT-1 (0.25 µM), LDN (0.2 µM), nicotinamide (10 mM), ILV (0.5 µM), glucose (18.4 mM), and vitamin C (0.25 mM) (**Figure 1J**).

### A revised protocol to generate GP2-positive pancreatic progenitors with trilineage potential

**Figure 2:**
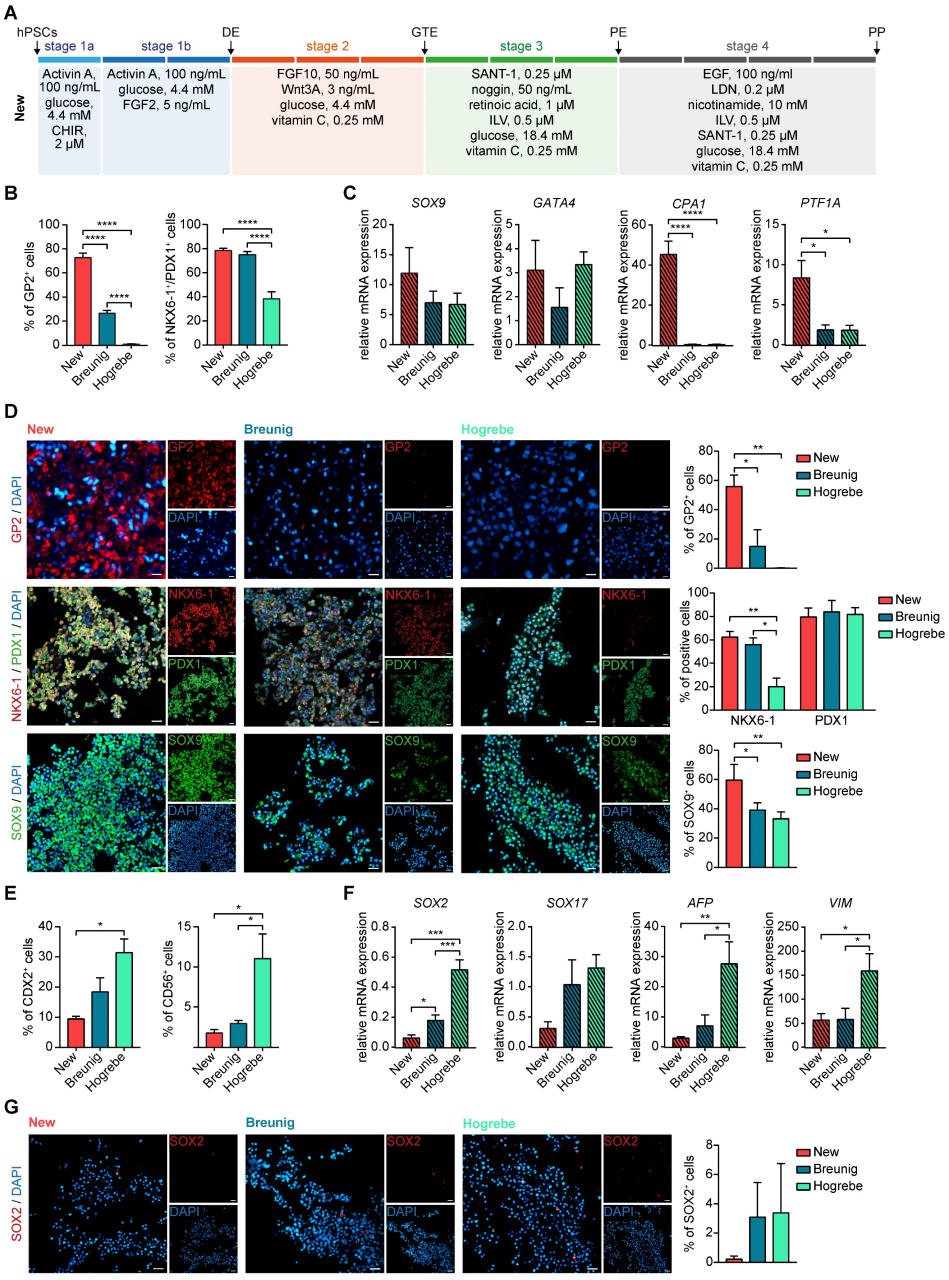
** The new protocol generates purer PPs with a higher number of multipotent GP2-positive progenitors.** (**A**) Optimized media composition was based on previous stage-specific compound testing. PPs generated with the new, Breunig 23, 24 and Hogrebe 14 differentiation protocol were characterized. PP markers were measured by (**B**) flow cytometry of GP2 (mean ± SEM, new n=15, Breunig n=15, Hogrebe n=7) and NKX6-1/PDX1 (mean ± SEM, new n=23, Breunig, n=22, Hogrebe n=11) and by (**C**) mRNA expression of *SOX9* (mean ± SEM, n=3*)*, *GATA4* (n=3), *CPA1* (n=3), and *PTF1A* (n= 4). (**D**) Representative immunofluorescence stainings of the PP markers GP2 (red), NKX6-1 (red) and PDX1 (green) and SOX9 (green) along with their quantification of positive cells normalized to DAPI (n=3). (**E**) Flow cytometry of the impurity markers CDX2 (mean ± SEM; n=4) and CD56 (n= 3). (**F**) Relative mRNA expression of the impurity markers *SOX2* (new n=4; Breunig n=4; Hogrebe n=3), *SOX17* (n=3), *AFP* (n=5)*,* and *VIM* (n=5) are shown (mean ± SEM. (**G**) Representative immunofluorescence stainings of the impurity marker SOX2 are shown and quantified (n=4). Counterstaining was performed with DAPI (blue). Scale bars of immunofluorescence images represent 50 µm. Gene expression levels in qPCR were normalized to the housekeeping gene *HMBS*. One-way ANOVA was used for evaluation of statistical significance. p < 0.05: *, p < 0.01: **, p < 0.001: ***. CHIR: CHIR99021; CPA1: carboxypeptidase 1; DE: definitive endoderm; GP2: glycoprotein 2; GTE: gut tube endoderm; hPSC: human pluripotent stem cell; ILV: Indolactam V; LDN: LDN-193189; PE: pancreatic endoderm; PP: pancreatic progenitor; VIM: vimentin

This systematic testing culminated in a new differentiation regimen powered to induce high yields of GP2-positive cells within the heterogeneous NKX6-1/PDX1 population and to suppress non-pancreatic lineages (**Figure 2A**). We next challenged our revised regimen against our previous protocol (Breunig; 23, 24) and a standard protocol in the field with proven endocrine effectiveness (Hogrebe; 14). Indeed, the GP2-positive cell fraction was strongly increased (~80%), with similar levels of NKX6-1/PDX1-positive cells (**Figure 2B, D, Figure S4A**). Also, the multipotent pancreatic progenitor markers SOX9, GATA4, carboxypeptidase 1 (CPA1) and PTF1A, which later get restricted to tip or trunk progenitors 1, 2, 38, showed significantly increased expression by the revised protocol (**Figure 2C-D**). Of note, the Hogrebe et al. protocol in our hands produced slightly lower proportions of NKX6-1^+^/PDX1^+^ cells on average, albeit close to the published efficiency 14 (**Figure 2B**). *Vice versa*, non-pancreatic cell contaminations (mesoderm, non-pancreatic endodermal progeny) were reduced accordingly on transcriptional but also on protein level when the GP2-optimized protocol was used (**Figure 2E-G, Figure S4A-B**), a key optimization to minimize potential perturbation in following endocrine or exocrine *in vitro* differentiations. Although most cross-lineage impurities were only slightly reduced, all markers showed the same trend, with the new protocol displaying the lowest amounts of impurities. The new protocol was also licensed for superior GP2 yields in a set of other (induced) pluripotent stem cell lines (iPSC: WTC-11; hESC: H1; **Figure S5A-B**).

**Figure 3:**
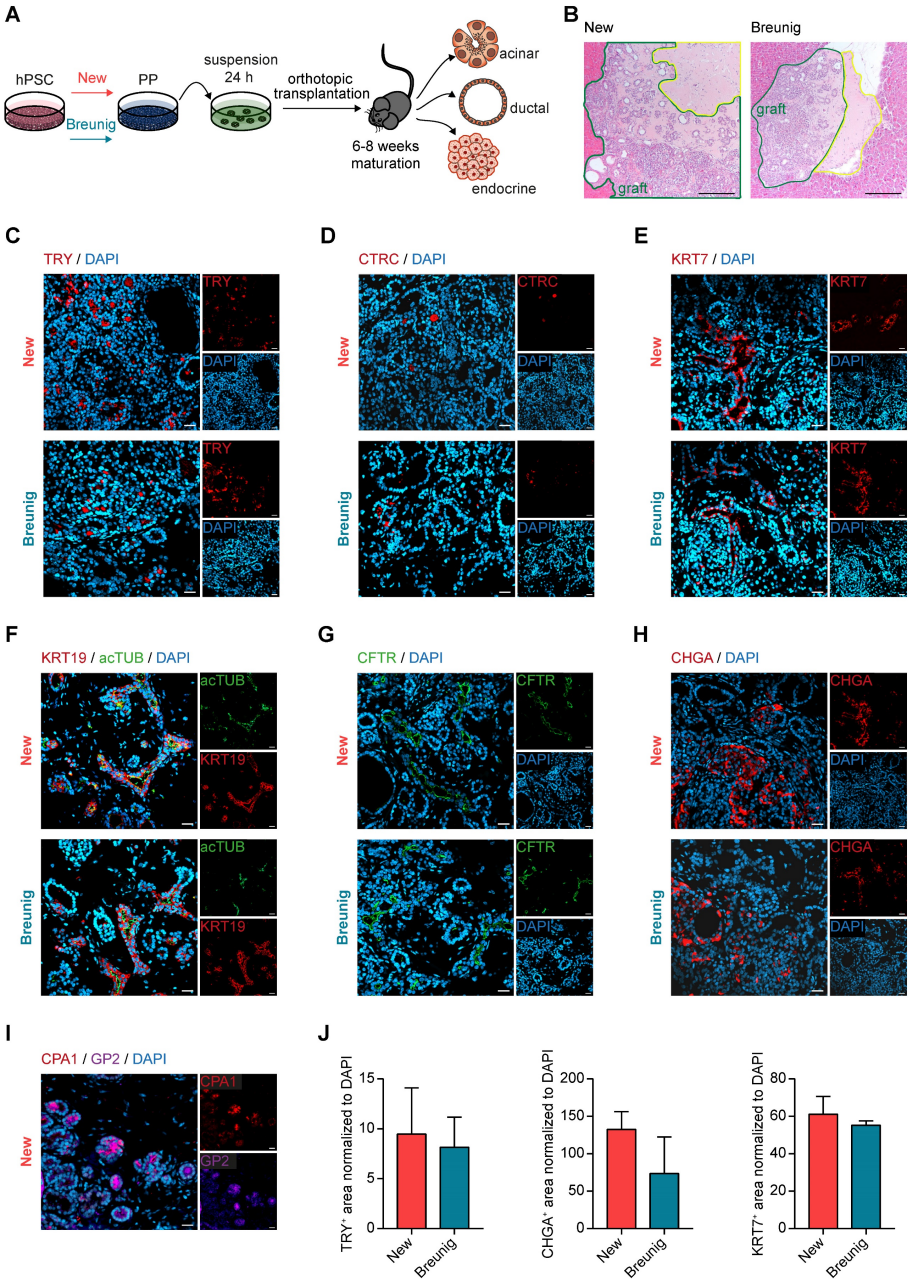
** New pancreatic progenitors mature into all pancreatic lineages upon *in vivo* transplantation.** (**A**) Workflow of orthotopic transplantation of PP spheres into NOD *scid* gamma (NSG) mice. (**B**) Hematoxylin and eosin staining of PP engraftment after 6 weeks of maturation in NSG mice. Grafts are bordered in green and the matrigel niche in yellow. Scale bars represent 200 µm. (**C-H**) Immunofluorescence staining of grafts after 6 weeks of maturation in NSG mice are shown. Representative images of the acinar markers (**C**) TRY (red) and (**D**) CTRC (red) are shown. Immunofluorescence stainings for the ductal markers (**E**) KRT7 (red), (**F**) KRT19 (red) co-stained with the cilia representing acTUB (green), and (**G**) CFTR (green). (**H**) Images are depicted for endocrine-specific CHGA (red). (**I**) Representative immunofluorescence image of CPA1 (red) and GP2 (purple) co-staining. Scale bars of immunofluorescence images represent 50 µm. (**J**) Quantification of TRY-, CHGA- and KRT7-positive area normalized to DAPI (n=3 grafts). acTUB: acetylated tubulin; CHGA: chromogranin A; CTRC: chymotrypsin C; hPSC: human pluripotent stem cell; KRT19: keratin 19; KRT7: keratin 7; PP: pancreatic progenitor; TRY: trypsin

Previously, enrichment of GP2 positive pancreatic progenitors by magnetic activated cells sorting (MACS) allowed to generate functional β-cells *in vivo* 34. In order to assess the developmental competence of our new PPs with high GP2 yield, we challenged their trilineage potential upon orthotopic transplantation into NOD *scid* gamma (NSG) mice in comparison to Breunig PPs (**Figure 3A**). Grafts were analyzed after 6 to 8 weeks and revealed the formation of fetal pancreas-like tissue (**Figure 3B**). Immunolabelling of the grafts identified mutually exclusive lineage segregation into morphologically distinct compact acinar structures positive for the digestive enzymes trypsin (TRY) and chymotrypsin C (CTRC) (**Figure 3C-D**), and tubular ductal structures expressing keratin 7 (KRT7) (**Figure 3E**). In line with previously reported *in vivo* expression patterns, GP2 localized to acinar cells and to not yet matured tip cells or PPs (**Figure 3I**) 33-35, 39. Ductal structures organized in keratin 19 (KRT19)-positive tubes and the maturity-indicating cilia were labelled by acetylated tubulin (acTUB) (**Figure 3F**). Proper segregation and maturation were further underpinned by the expression of CFTR restricted to the ductal compartment (**Figure 3G**). During human embryonic pancreatogenesis, the first endocrine clusters are visible from 10 weeks on, followed by the structural organization in islets from 12 weeks post-conception (wpc) 1, 40-43. Indeed, chromogranin A (CHGA)-positive endocrine cells could be detected in PP grafts that were already organized in islet-like clusters (**Figure 3H).** Since even PDX1 positivity alone defines the pancreatic lineage 7, 29, 44, 45, PDX1-positive pancreatic epithelium has already the capability for proper maturation and lineage segregation *in vivo*, due to a supportive and stimulative environment from the murine pancreas (data not shown). Therefore, subsequent quantifications of the respective immunofluorescence signals did not reveal significant differences between the protocols, albeit there was a trend of improved maturation for the new protocol in all three lineages (**Figure 3J**).

### GP2 expression levels and lineage potential in pancreatic progenitors

**Figure 4:**
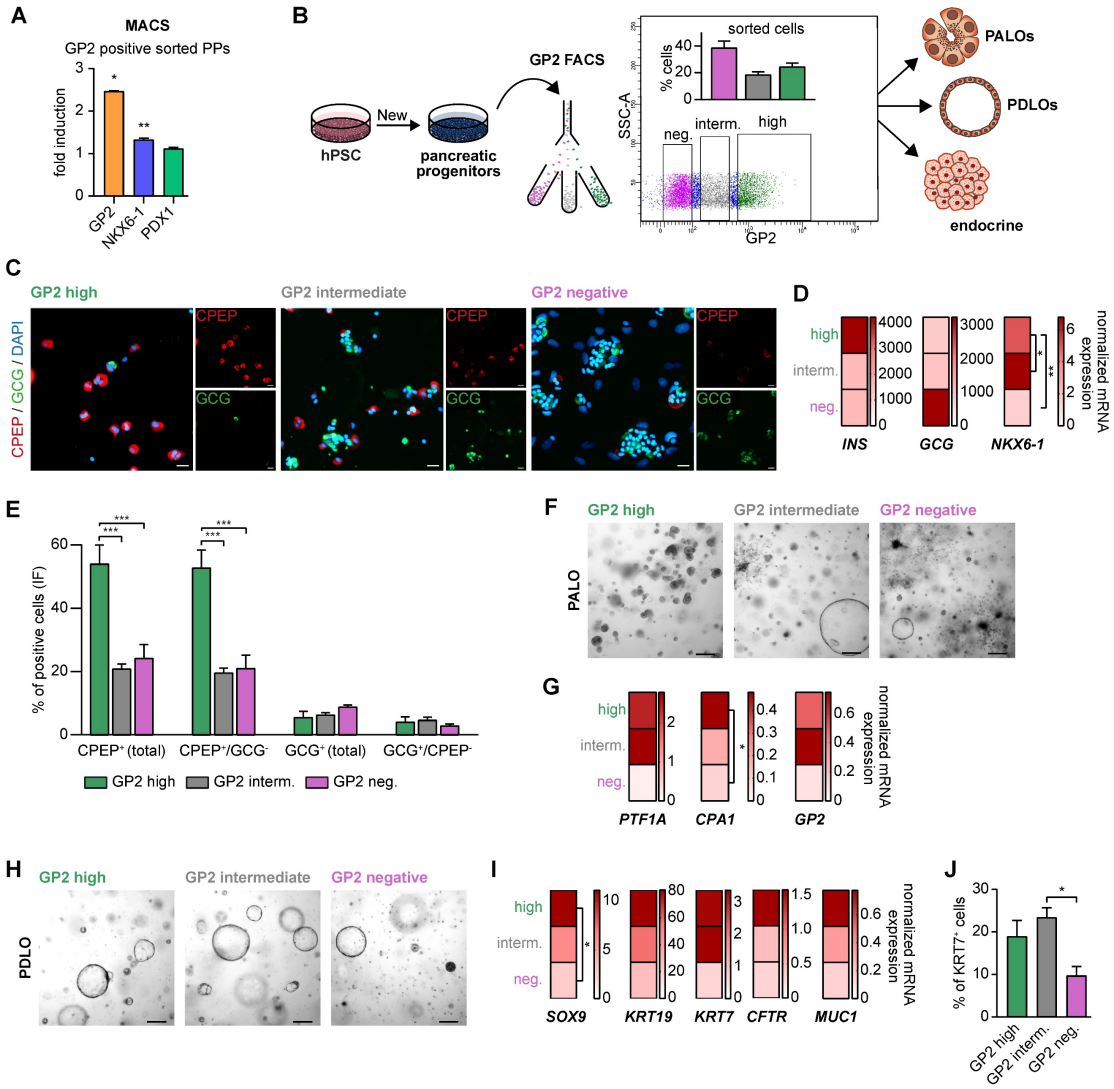
** GP2-enriched pancreatic progenitors improve trilineage differentiation capacity *in vitro*.** (**A**) Magnetic-activated cell sorting (MACS) of pancreatic progenitors and subsequent flow cytometry of GP2 or NKX6-1 and PDX1 labeled cells of the unsorted and GP2 positive sorted population. FC results are shown as fold induction over unsorted PPs (mean ± SEM, n=3). (**B**) Schematic overview of fluorescence activated cell sorting (FACS) of GP2 labeled PPs, followed by acinar, ductal, and endocrine differentiation of GP2 high, intermediate and negative sorted populations. (**C**) In-well staining of endocrine cultures derived from GP2 high, intermediate and negative populations with endocrine markers CPEP (red) and GCG (green). Scale bars represent 50 µm. (**D**) Relative mRNA expression of *INS*, *GCG* and *NKX6-1* for GP2 high, intermediate and negative populations are shown as heatmaps (mean, n=3). (**E**) Quantification of immunofluorescence staining of n=6 fields of view. (**F**) Brightfield images of acinar cultures derived from GP2 high, intermediate and negative populations. Scale bars represent 200 µm. (**G**) Relative mRNA levels of *PTF1A*, *CPA1*, and* GP2* are depicted for PALOs generated of GP2 high, intermediate or negative PPs (n=4). (**H**) Representative brightfield images of PDLOs generated from GP2 high, intermediate, or negative PPs. Scale bars represent 200 µm. (**I**) Relative mRNA expression levels of *SOX9, KRT19, KRT7, CFTR*, and *MUC1* are depicted as heatmaps (n=4). (**J**) FC analysis at the PDLO stage shows KRT7 positive cells of GP2 high, intermediate or negative sorted populations (n=4). All gene expression levels are depicted as mean and were normalized to *HMBS*. Statistical analysis was performed with ordinary one-way ANOVA. AMY2A: amylase 2A; CPA1: carboxypeptidase-1; CPEP: c-peptide; GCG: glucagon; GP2: glycoprotein 2; IF: immunofluorescence; INS: insulin; KRT19: keratin 19; KRT7: keratin 7; PALO: pancreatic acinar-like organoid; PDLO: pancreatic duct-like organoid

First, GP2-based MACS confirmed that its enrichment was accompanied by a significant increase in NKX6-1 in the homogenous PDX1 positive population (**Figure 4A**, 35). This confirms GP2 as valid PP marker for an NKX6-1 positive subset suitable to resolve the heterogeneity of PP subpopulations. To further dissect the biology of GP2 expressing cells, we stratified GP2 expression into “high”, “intermediate” and “negative” via fluorescence-activated cell sorting (FACS) and challenged the subsequently purified GP2^high^, GP2^intermediate^ and GP2^negative^ PP populations for their endocrine, ductal, and acinar differentiation capacities (**Figure 4B**). In terms of endocrine differentiation ability, the GP2^high^ population showed tremendous superiority in the formation of β-like cells, expressing higher yields of β-cell specific markers at protein and mRNA level (**Figure 4C-E**). The GP2^negative^ population appeared to be more prone to generate α-like cells (**Figure 4D**). These observations are concordant with published α-cell protocols, bypassing the expression of the typical PP marker NKX6-1 46.

Further, FACS-purified GP2^high^, GP2^intermediate^ and GP2^negative^ cells were also directed towards the acinar lineage 25. Only from the GP2^high^ fraction, we observed a morphologically homogenous culture with structures resembling acinar morphology, while the other fractions resulted in heterogeneous cultures with overgrowing cyst- and fibroblast-like structures (**Figure 4F**). Accordingly, there was a strong induction of various acinar markers such as *PTF1A* and* CPA1* in the GP2^high^ progeny (**Figure 4G**).

During ductal differentiation the GP2^negative^ population was completely inefficient as judged by morphology, qPCR, and FC for KRT7 (**Figure 4H-J**). The GP2^high^ population was superior in the expression of duct-specific markers, such as *SOX9*, *KRT19*, *KRT7*, *CFTR*, and *MUC1*. The significantly higher protein levels of KRT7 in the GP2^high^ and GP2^intermediate^ populations further confirmed the enhanced ability of GP2-enriched populations in proper ductal maturation.

### Superior endocrine and ductal differentiation from GP2-enriched pancreatic progenitors

**Figure 5:**
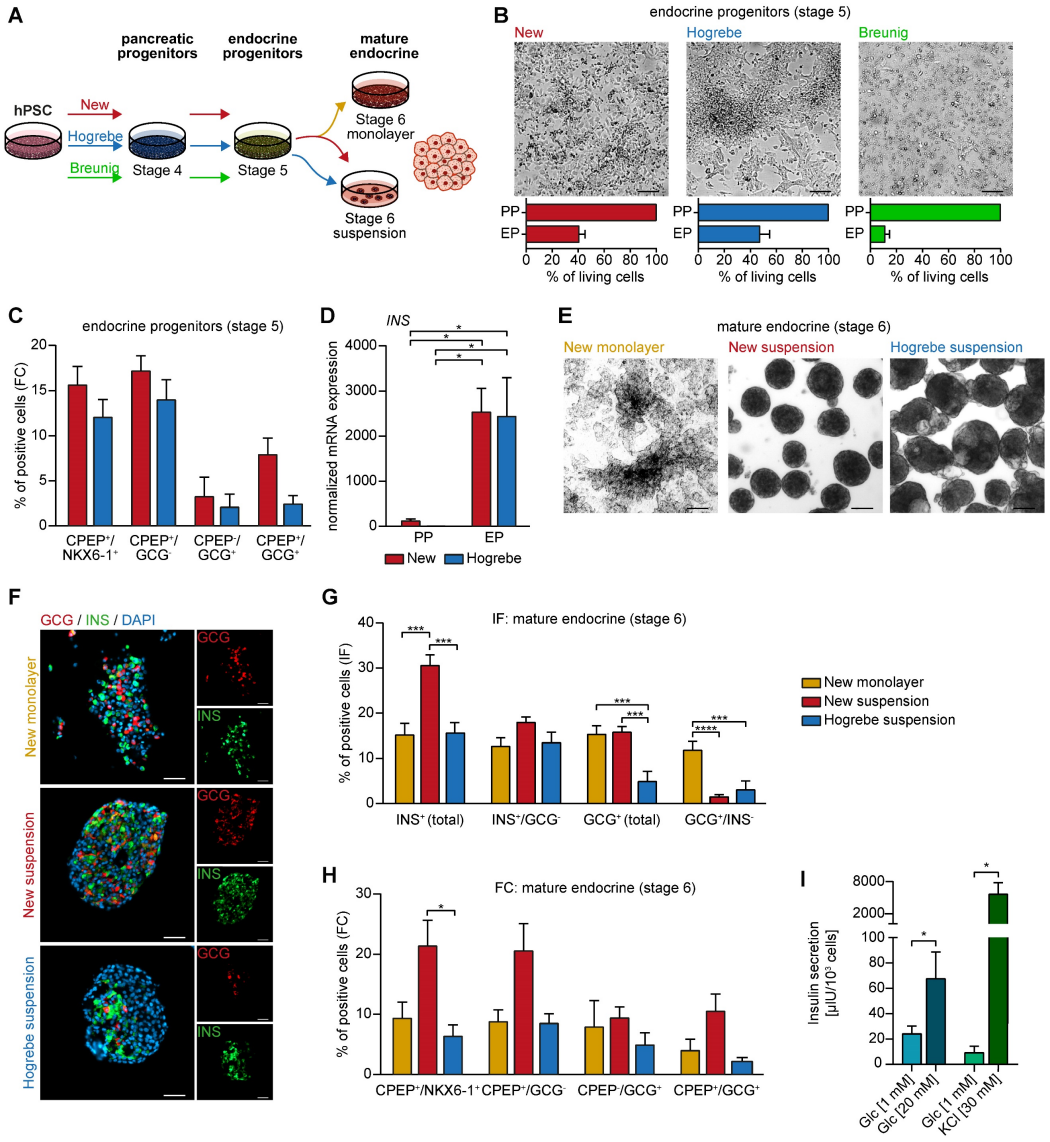
** New pancreatic progenitors differentiate into the endocrine lineage *in vitro*.** (**A**) Workflow of PP differentiation according to the new, Hogrebe 14, and Breunig 23, 24 protocol. PPs were further differentiated according to the endocrine monolayer protocol published by Hogrebe et al. 14 for 14 days, followed by another 7 days in either planar or suspension culture. (**B**) Brightfield images of the endocrine stage reflect different cell densities after 21 days of differentiation supported by cell counting and the resulting percentage of living cells compared to the PP stage (n=3). Scale bars represent 200 µm. (**C**) Flow cytometry (FC) analysis at the endocrine progenitor stage showing NKX6-1, CPEP, and GCG positive populations (n=7). (**D**) Relative mRNA expression of *INS* at the endocrine progenitor stage (EP) compared to pancreatic progenitors (PP) is depicted. Gene expression levels were normalized to the housekeeping gene *HMBS* (n=3). (**E**) Representative brightfield images of mature endocrine cells (stage 6) in monolayer or suspension culture. (**F**) Immunofluorescence stainings of GCG (red) and INS (green) are shown for mature endocrine cells along with their (**G**) quantification (new monolayer n=17, new suspension n=21, Hogrebe suspension n=16, field of views). Scale bars represent 100 µm. (**H**) FC results of CPEP, NKX6-1 and GCG are depicted (new monolayer n=4, new suspension n=6, Hogrebe suspension n=4). (**I**) Glucose or KCl-stimulated insulin secretion was performed with mature endocrine cells generated with the new monolayer protocol. Secreted insulin was measured by ELISA (n=9). Bar graphs depict mean ± SEM. Statistical analysis was performed with ordinary one-way ANOVA or two-tailed t-test, p < 0.05: *, p < 0.01: **, p < 0.001: ***. CPEP: c-peptide; GCG: glucagon; hPSC: human pluripotent stem cell; INS: insulin; PP: pancreatic progenitor

Next, we wanted to challenge our new PP protocol to allow lineage-controlled and simultaneous differentiation into either endocrine or ductal cells from the same PP culture. First, we induced PPs according to our new protocol, Hogrebe et al. 14 or Breunig et al. 23, 24, followed by stepwise endocrine lineage induction as recently reported 14 (**Figure 5A**). Interestingly, differentiation of Breunig PPs caused excessive cell death, while PPs derived with the new protocol formed cell clusters resembling NKX6-1/C-peptide (CPEP) double positive endocrine progenitor cells at stage 5 in comparable numbers to Hogrebe PPs 14 (**Figure 5B-C, Figure S6A-B**). Insulin (*INS*) mRNA expression levels confirmed these findings (**Figure 5D**). The differentiation efficiency could be reproduced in an additional iPSC line (**Figure S5C**). Further endocrine enrichment and maturation was achieved by applying enriched serum free medium (ESFM) for 14 days either in monolayer or suspension culture (**Figure 5E**). Immunofluorescence staining complemented by flow cytometry analysis revealed a significant increase of NKX6-1/CPEP as well as INS (up to 30%) expressing cells (**Figure 5F-H, Figure S6A**). Mature endocrine cells entirely generated in monolayer with the new protocol produced more monohormonal glucagon (GCG)-positive cells. While the additional aggregation step led to a higher number of INS- and CPEP-producing monohormonal β-cells, it also generated slightly more bihormonal CPEP- and GCG-double positive cells (**Figure 5F-H**). Finally, we functionally challenged the resulting β-like cells by performing a glucose-stimulated insulin secretion (GSIS) assay or stimulation with KCl to confirm the generation of proper β-like cells *in vitro* (**Figure 5I**).

**Figure 6:**
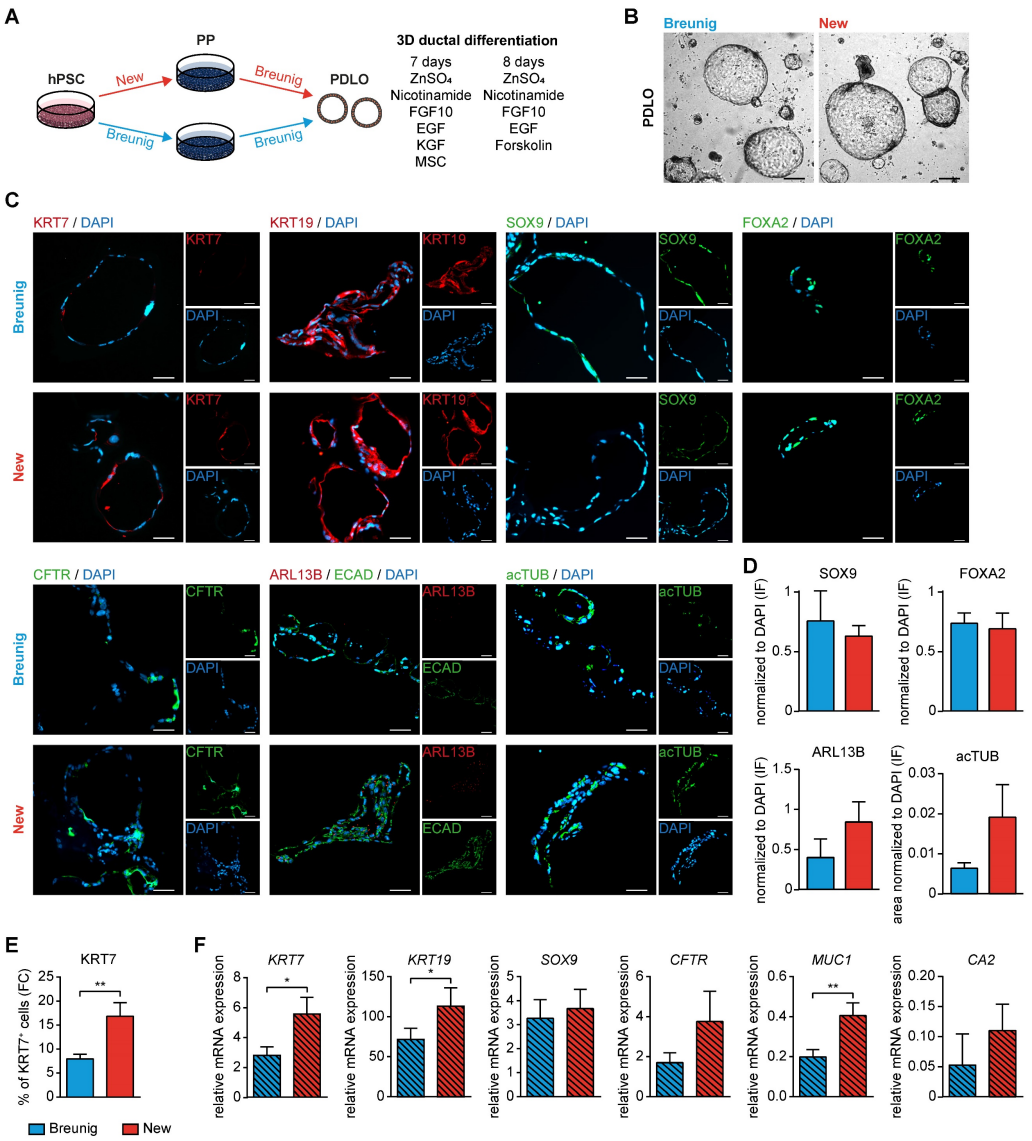
** Improved *in vitro* ductal differentiation of new pancreatic progenitors.** (**A**) Schematic overview of pancreatic progenitor (PP) differentiation with the new and Breunig protocol 23, 24 and further ductal commitment according to our published protocol with minor modifications 23, 24. (**B**) Brightfield images of pancreatic duct-like organoids (PDLOs) at day 28. (**C**) Representative immunofluorescence staining at the PDLO stage of ductal markers KRT7 (red), KRT19 (red), SOX9 (green), FOXA2 (green), CFTR (green), ARL13B (red), ECAD (green), and acTUB (green). Cells were counterstained with DAPI (blue). (**D**) Immunofluorescence stainings were quantified by normalizing the number of SOX9 (new n=7, Breunig n=4), FOXA2 (new n=9, Breunig n=4), ARL13B (new n=8, Breunig n=5) positive structures and acTUB positive area (new n=3, Breunig n=3) to DAPI positive structures. N represents fields of view. (**E**) Flow cytometry results of KRT7 (n=10) and (**F**) qPCR results of the ductal markers *KRT7* (n=9), *KRT19* (n=9), *SOX9* (n=9), *CFTR* (n=9), *MUC1* (n=9)*,* and* CA2* (n=3) are shown. All gene expression data were normalized to the housekeeping gene *HMBS*. Bar graphs depict mean ± SEM. Significance was evaluated with paired two-tailed t-test, P < 0.05: *, p < 0.01: **, p < 0.001: ***. Scale bars of bright-field and immunofluorescence images represent 200 µm and 100 µm, respectively. GP2: glycoprotein 2; hPSC: human pluripotent stem cell; KRT19: keratin 19; KRT7: keratin 7

Second, the various PP types were directed towards the ductal lineage following our recently published protocol 23, 24 (**Figure 6A**). Morphologically, the pancreatic duct-like organoids (PDLOs) generated from the new PPs were similar in shape and size with a ring-shaped and single-layered organoid morphology in a virtually pure culture as reported before (**Figure 6B**) 23, 24. The homogenous expression of KRT19*,* SOX9 and FOXA2 properly licensed ductal lineage entry (**Figure 6C**), which was also reproducible in an additional cell line (**Figure S5D**). Usually, the maturation level of stem cell-derived progeny is limited to a fetal state without reaching full maturation 47-50. KRT7, a key ductal maturity marker, was increased both on the protein level (up to 20% KRT7 positive cells) and on the mRNA level (**Figure 6C, E-F**). We further substantiate these maturation improvements in the PDLOs generated with our new protocol by assessing the expression of additional mature ductal markers on protein (CFTR, ARL13B, acTUB) and on transcriptional level (*CFTR*; *carbonic anhydrase* (*CA2*)) (**Figure 6C-D, F**)**.** Importantly, another essential maturation marker, *mucin 1* (*MUC1*), and even the widely expressed ductal marker KRT19, were significantly upregulated in the new protocol, again supporting the improved maturation.

### GP2-enriched PPs can be stored without losing ductal lineage potential

Since ductal lineage specification takes almost a month in total, we aimed for more experimental flexibility by starting from an intermediate stage of the new protocol in stock. Therefore, we induced pancreatic endoderm cells according to **Figure 2** and stored them in liquid nitrogen (**Figure S7A**). After thawing, the new stage 4 was completed to generate PPs, followed by PDLO differentiation. Intriguingly, frozen PE cells preserved their differentiation capacity, giving rise to an NKX6-1 and GP2-high cell population and to PDLOs with similar ring-like morphology independent of the freeze-thaw cycle (**Figure S7B-C**). Interestingly, KRT7 together with a set of additional maturation markers (CFTR, *MUC1*) could be further increased when frozen PE cells were subsequently differentiated (**Figure S7D-F**).

### Purification of GP2^high^ PPs allows the generation of pancreatic acinar-like organoids *in vitro*

**Figure 7:**
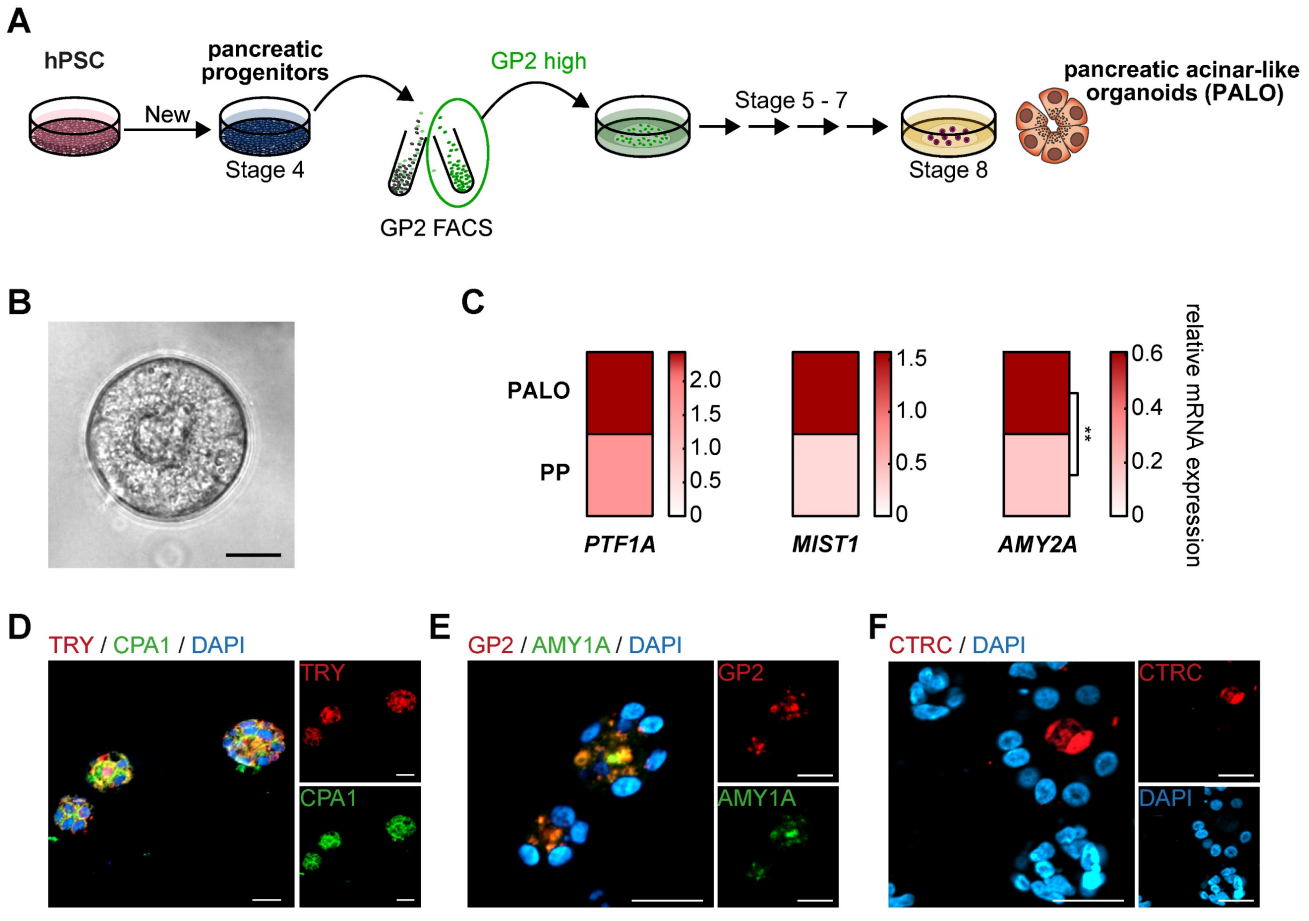
** GP2-enriched progenitors are a prerequisite to generate pancreatic acinar-like organoids *in vitro*.** (**A**) Schematic illustration to generate pancreatic acinar-like organoids (PALOs) derived from human pluripotent stem cells (hPSC), highlighting a GP2 FACS purification step at the pancreatic progenitor (PP) stage. The GP2 high sorted population of PPs was further challenged to the acinar direction 25. (**B**) Brightfield image of PALO at stage 8. Scale bar represents 20 µm. (**C**) Mean relative mRNA expression values of acinar markers *PTF1A*, *MIST1*, and *AMY2A* are shown as heatmap for PALOs and PPs (n=4). The *PTF1A* expression data of PALOs are the same as the corresponding GP2^high^ values in **Figure 4G**. Significance was evaluated with two-tailed t-test, p < 0.05: *, p < 0.01: **, p < 0.001: ***. Immunofluorescence images of typical acinar markers (**D**) TRY (red) and CPA1 (green), (**E**) GP2 (red) and AMY1A (green), and (**F**) CTRC (red) are shown. Scale bars represent 20 µm. AMY1A: amylase 1A; AMY2A: amylase 2A; CPA1: carboxypeptidase 1; CTRC: chymotrypsin C; FACS: fluorescence-activated cell sorting; GP2: glycoprotein 2; TRY: trypsin

Based on the acinar differentiation of sorted PPs with varying levels of GP2 expression, we fine-tuned the protocol by permanently including a GP2^high^ FACS-purification step at the PP stage (**Figure 7A**). This modification allowed the generation of organoids with clear acinar morphology and upregulation of acinar marker transcripts such as the digestive enzyme *amylase* and the transcription factor *MIST1*, both being hallmarks of acinar identity 51-53 (**Figure 7B-C**). Immunostaining of these pancreatic acinar-like organoids (PALOs) confirmed protein expression of further digestive enzymes residing to the apical, secretion side indicating a true acinar fate, i.e. TRY, CTRC and amylase (**Figure 7D-F**).

### Single-cell RNA sequencing reveals strong lineage heterogeneity across pancreatic progenitors

**Figure 8:**
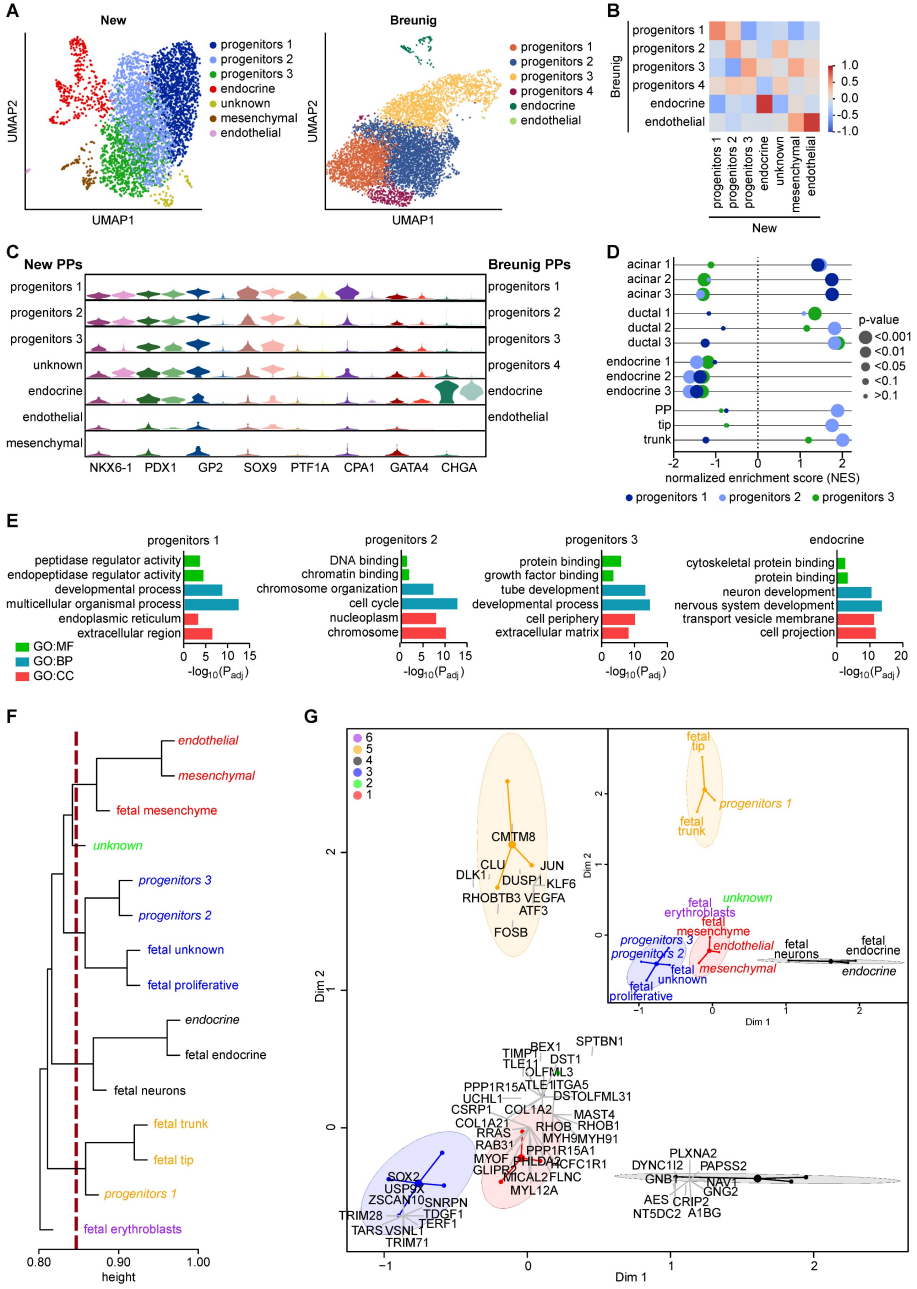
** scRNA-seq reveals heterogeneity in pancreatic progenitors which cluster close to a human fetal pancreas dataset.** (**A**) Dimension reduction with UMAPs representing new and Breunig PPs 54 in 7 and 6 cell clusters, respectively. (**B**) Cluster-specific Pearson correlation of new and Breunig PPs resembling only weak correlation between progenitor clusters. (**C**) Violin plots representing expression patterns of selected PP markers used for cluster annotation. Darker and lighter colors indicate subclusters from new PPs or Breunig PPs, respectively. (**D**) Preranked gene set enrichment analysis (GSEA, Broad Institute, 91) of new progenitors 1, 2, and 3 against the following datasets: acinar 1 (acinar subpopulation, Krentz et. al. 93), acinar 2 (acinar subpopulation, Bastidas-Ponce et al. 94), acinar 3 (MsigDB C8.all., Descartes fetal pancreas acinar cells 91, 92), ductal 1 (ductal subpopulation, Krentz et el. 93), ductal 2 (ductal subpopulation, Bastidas-Ponce et al. 94), ductal 3 (MsigDB C8.all., Descartes fetal pancreas ductal cells 91, 92), endocrine 1 (CHG subpopulation, Krentz et al. 93), endocrine 2 (endocrine subpopulation, Bastidas-Ponce et al. 94), endocrine 3 (MsigDB C8.all.v7.5.1, Descartes fetal pancreas islet endocrine cells 91, 92), PP, tip, trunk (PP, tip, and trunk subpopulations, all Bastidas-Ponce et al. 94). (**E**) Depiction of relevant GO terms of GO:MF (molecular function), GO:BP (biological process), and GO:CC (cellular component) among the top 7 significantly enriched terms for progenitors 1, 2, 3, and endocrine cluster. (**F**) Hierarchical clustering of the new PP clusters with a human fetal pancreas dataset (7-10 wpc) of Gonçalves et al. 60. New PP clusters are drawn in italic. (**G**) Correspondence analysis results in clustering and annotated genes that can be overlaid with the results of the hierarchical clustering. CPA1: carboxypeptidase 1; CHGA: chromogranin A; GO: gene ontology; GP2: glycoprotein 2; NES: normalized enrichment score; P_adj_: adjusted p-value

To interrogate the transcriptional landscape underlying our GP2-enriched pancreatic progenitor cell population, we performed single-cell RNA sequencing (scRNA-seq). Principal component analysis for dimensionality reduction, Leiden clustering, and Uniform Manifold Approximation and Projection (UMAP) allowed the visualization of 7 transcriptionally distinct clusters in the new protocol, which were compared to the PP clusters of the Breunig et al. protocol 54 (**Figure 8A, Figure S8**). Progenitor cluster 1 contained cells expressing markers of the tip domain (e.g., *GATA4*, *CPA1*, *PTF1A*) and was further enriched for the expression of multipotency markers (*GP2*, *NKX6-1*, *PDX1*, *SOX9*) and the acinar markers *PRSS1* and *PRSS2*. While clusters 2 and 3 both expressed high levels of the common pancreatic lineage marker *PDX1*, they differed in *NKX6-1*, *SOX9*, and *CPA1*, with lower expression in progenitor cluster 3 (**Figure S9, Table S7**). To globally compare both PP single-cell datasets, we performed pairwise Pearson correlation analysis across the identified clusters (**Figure 8B**). Interestingly, high correlations were only observed in the endocrine as well as the endothelial cell cluster, while the various progenitor clusters showed only moderate correlation. The inter-cluster heterogeneity was further highlighted upon selective comparison of *GP2*, *PTF1A*, *CPA1*, and *GATA4* together with *NKX6-1*, *PDX1,* and *SOX9* suggesting relevant enrichment for acinar- or tip-directed cell types in progenitor cluster 1 (**Figure 8C, Figure S9**). To globally assess whether distinct PP clusters resemble specific lineage fates, we performed gene set enrichment analysis (GSEA) with acinar, ductal, endocrine, and progenitor gene sets (**Table S8**): Here, acinar genes showed a positive normalized enrichment score (NES) for the new progenitor cluster 1, while ductal genes were enriched in cluster 2 and 3 (**Figure 8D**). Progenitors 2 also displayed enrichment in PP, tip, and trunk gene sets, with the latter also arising in progenitor cluster 3. Unbiased gene ontology (GO) term analysis confirmed the acinar fate of cluster 1, with enrichment of terms such as “peptidase regulator activity” or “endoplasmic reticulum”, likely linked to acinar exocytosis of digestive enzymes 55-57 (**Figure 8E**). In contrast to cluster 3 showing enrichment for terms such as “tube development” 58 and “extracellular matrix” 59 relevant for ductulogenesis, the progenitor cluster 2 was enriched for e.g. “DNA binding” and “cell cycle” indicated active proliferation and expansion in pancreatic progenitors 3. From the marker profile, we concluded that PP cluster 2 might most closely resemble multipotent progenitors while cluster 3 might harbor a ductal and cluster 1 an acinar lineage bias. Next, we mapped the PP clusters into a human fetal pancreas (7-10 wpc) single-cell dataset 60. Correlation analysis revealed that progenitors 1 tightly correlated with the human fetal trunk and tip clusters, while progenitors 2 and 3 more closely resembled the fetal proliferating population (**Figure 8F**). Further, the endocrine cluster correlated with fetal neuronal and endocrine cells, and the endothelial and mesenchymal clusters closely resembled fetal mesenchyme. Correspondence analysis was able to replicate the clustering (**Figure 8G**). The newly identified clusters can thus be superimposed and defined with the top genes of the correspondence analysis serving as potential markers for further identification of specific clusters within a distinct progenitor population (**Figure 8G, Figure S10, Table S9**). The fetal tip and trunk clusters correlating with progenitor cluster 1 were defined by transcription factors involved in MEK/ERK signaling in acinar cells (JUN1, DUSP1, FOSB) 61, also known as early response genes, rapidly induced by extracellular stimuli, such as EGF 62. VEGFA, in turn, is important for epithelial-endothelial crosstalk in trunk cells 63. Further, RHOBTB3, a protein required for vesicle transport from endosome to Golgi 64, and DLK1, a NOTCH ligand 64, were expressed. Markers specific for cell cycle regulation and proliferation, such as the E3-ubiquitine ligases TRIM71 and TRIM28 65 and the telomere protein TERF1 66, defined progenitor clusters 2 and 3 and fetal proliferative and unknown. Additionally, this cluster was defined by SOX2 67 and the pluripotency markers TDGF1 68, and ZSCAN10 69.

### Identifying pathways that affect lineage heterogeneity in pancreatic progenitors

**Figure 9:**
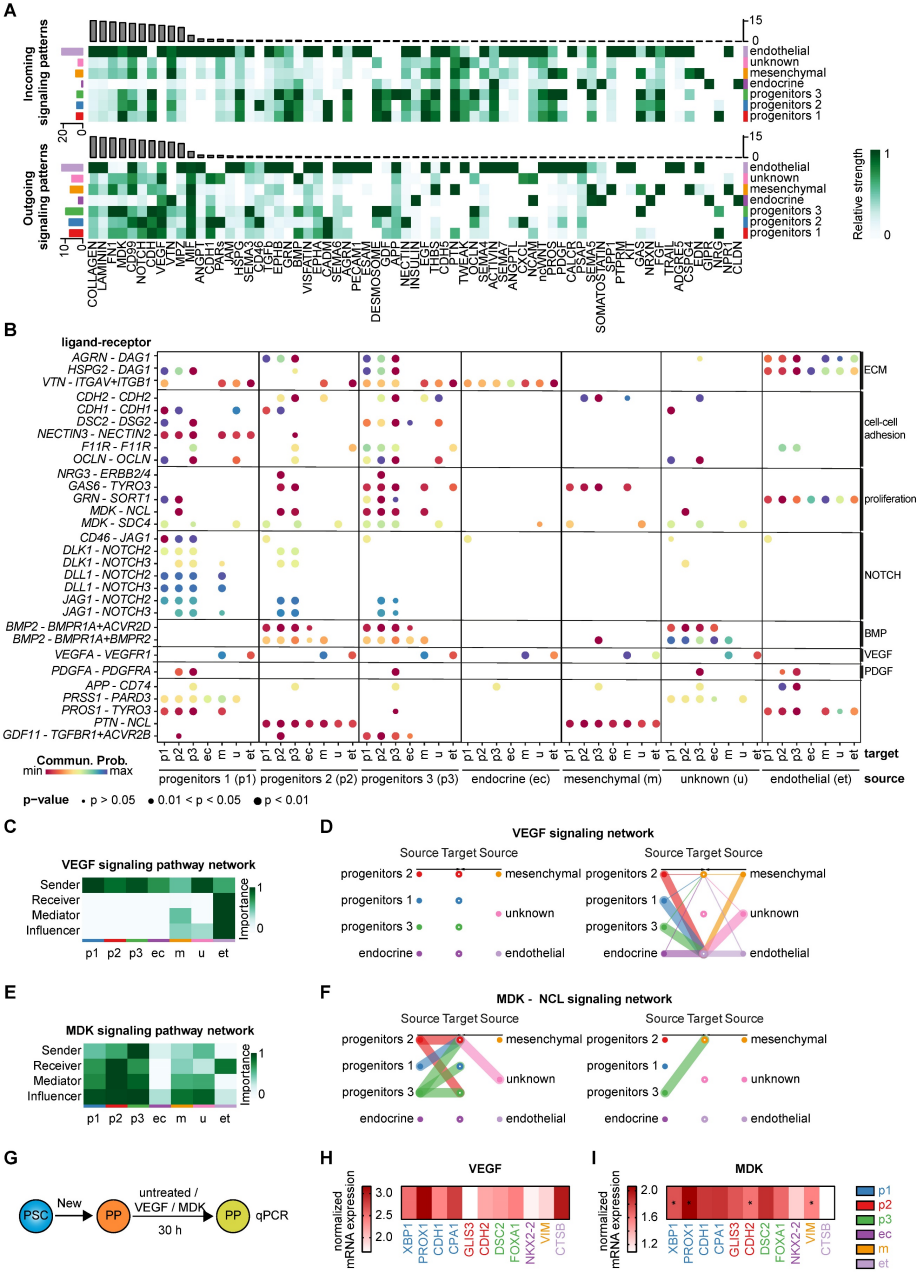
** Identified ligand-receptor signaling networks in heterogeneous pancreatic progenitors.** (**A**) Overview of significant incoming and outgoing signaling flow patterns across the 7 different pancreatic progenitor (PP) clusters. The heatmaps show the signal intensity (relative strength) of each pathway in each cell type for outgoing or incoming signaling. (**B**) Bubble plot representation of selected ligand-receptor interactions and their relationship between the individual cell clusters, defined as source and target. Dot color represents communication probability and dot size represents significance indicated by p-value. Normalization was performed line-wise within a ligand-receptor pair. (**C**) The cluster-specific role identification - sender, receiver, mediator, influencer - in VEGF signaling is depicted as heatmap. (**D**) Intercellular communication network of VEGF signaling is presented as hierarchy plot. Communication probability is reproduced by the line thickness. (**E**) Heatmap representing the cluster-specific role in MDK signaling pathway. (**F**) Hierarchy plot of MDK-NCL signaling network. (**G**) Schematic illustration of compound treatment at the pancreatic progenitor stage. (**H**) Mean gene expression data of cluster-specific markers for VEGF treated PPs are shown (n=5 differentiations). Relative mRNA expression values were normalized to untreated cells. (**I**) Mean gene expression data of MDK-treated PPs normalized to untreated cells are depicted as heatmap (n=5 differentiations). Cluster-specific markers are shown. Significance was evaluated with two-tailed t-test, p < 0.05: *, p < 0.01: **, p < 0.001: ***. MDK: midkine; NCL: nucleolin; VEGF: vascular endothelial growth factor

To decipher the cell-cell interactions within and between the identified clusters, we performed a comprehensive ligand-receptor analysis using the tool CellChat 70. The high heterogeneity of pancreatic progenitors was again reflected in the diversity of cell-cell interactions across various clusters (**Figure S11A-B**). Top ligand-receptor (L-R) hits in cell-cell signaling highlighted the importance of the extracellular matrix, such as collagen or laminin, at this stage of maturation dominated by the endothelial cluster (**Figure 9A, Figure S11C**). Incoming and outgoing signaling cues are mainly associated with ECM components and cell-cell adhesion molecules. Communication from and to endothelial cells was pronounced across all three progenitor clusters indicating the relevance of vascularization for proper pancreatic development 71. Progenitors 1-3 were mainly involved in cell adhesion, as suggested by factors such as CDH, CADM, Desmosome, GDF, and FN1, for both incoming and outgoing signaling. Cell growth- and proliferation-specific signaling cues were also pronounced within the progenitor clusters. Overall, midkine (MDK), Notch, and VEGF signaling determined a large set of signaling cues in progenitors (**Figure 9A**). The endocrine cluster was less involved in ECM signaling pathways than any other cluster (**Figure 9A**), which may explain the efficient *in vitro* generation of endocrine cells in matrix-free suspension cultures 9, 36.

### Distinct ligand-receptor interactions specify the progenitor subpopulations

We selected interesting ligand-receptor pairs underpinning again the importance of ECM components for progenitor populations (**Figure 9B**). Cell-cell adhesion molecules such as Cadherin-1 and -2 were predominantly found in the progenitor populations (**Figure S11D**). Interestingly, the ligand DSC2, recently published as a relevant signaling cue for endoderm formation 72, was mainly expressed in ductal-like progenitors 3, transmitting signals to progenitor 1-3 and endocrine clusters via the DSG2 receptor (**Figure S11E**). Proliferative cues mostly originated from progenitor cluster 3 which was exemplified by selected ligand-receptor pairs (GAS6-TYRO3, MDK-NCL) (**Figure S11F**) 73. Outgoing GAS6 signaling predominantly occurred from progenitor cluster 3 to receiving progenitors 2 and 3. In addition, JAG1 also served as receptor for CD46 signaling, where the ligand CD46 mainly transmitted the signal to progenitor cluster 1 (**Figure S11G**). Progenitors 1 and to some extent progenitors 2 were identified as the major senders of NOTCH ligands (DLK1, DLL1, and JAG1) underlining the importance of the NOTCH signaling pathway in pancreatogenesis (**Figure S11H**) 74-76. Previously known signaling pathways, such as BMP, PDGFA, and EGF were re-identified in our analysis 60, 77.

BMP signaling mainly occurred from progenitors 2 and 3 through signaling via BMP2 to the progenitor 1-3 and the endocrine cluster, receiving the signal via BMP and Activin receptors (BMPR1A, ACVR2D, BMPR2) (**Figure 9B**). Outgoing signaling via PDGFA was primarily driven by endothelial cells, whereas incoming signaling via PDGFR was mostly found in progenitors 3 and partially in progenitors 2. Other interesting and more cluster-defining/-specific receptor-ligand interactions include the PAR signaling pathway network involving the acinar-specific ligand PRSS1 78 of progenitors 1, mainly signaling to the endothelial cluster through the PARD3 receptor (**Figure S11I**), and pleiotrophin-nucleolin (PTN-NCL) in progenitors 2 (**Figure S11J**). GDF signaling was predominantly mediated by the ligand GDF11 from progenitors 3, whereas incoming signaling was received from progenitors 1, 2, and 3 via TGFβ and activin receptors. GDF11 has been described to promote exocrine pancreatic tissue growth (**Figure S11K**) 79.

For validation of the *in silico* defined ligand-receptor interactions, we treated pancreatic progenitors for 30 h with the respective compounds (VEGF or midkine) or control (untreated) (**Figure 9G**). We analyzed the gene expression of cluster-specific transcripts to examine the effect of the respective compounds on each cluster.

VEGF signaling is most important for epithelial-endothelial crosstalk which is also reflected in the* in silico* ligand-receptor analysis (**Figure 9C-D**) 63, 71. Epithelial clusters, such as the progenitor clusters, were the main transmitters, whereas the endothelial cluster was the only receiver in VEGF signaling. To validate these predicted LR interactions, we treated the PPs with VEGFA and assessed the mRNA levels of certain cluster-specific markers (**Figure 9H**). The results were closely resembling the *in silico* LR predictions, as transcripts specific for progenitors 1 and the endothelial cluster were upregulated.

The MDK signaling pattern was transduced by the ligand midkine of progenitor cluster 3, with progenitor cluster 2 and 3 being the major recipients via the receptor NCL (**Figure 9E-F**). A truncated isoform of the ligand midkine was previously found to be expressed throughout the E11 murine pancreatic epithelium 80. To experimentally validate the ligand-receptor interactions predicted *in silico*, we treated the PPs with human recombinant midkine. We examined the mRNA expression levels of markers defining the different clusters (**Figure 9I**). Only 30 h of treatment with midkine resulted already in an increase in progenitor 1, 2 and 3 specific markers, which are the major clusters predicted to be involved in midkine signaling at the PP stage.

## Discussion

Mimicking fetal pancreas development *in vitro* is to date mainly biased to the generation of glucose-responsive β-cells. Although we and others have recently published protocols for ductal differentiation 23-25, the PPs generated with distinct approaches are already primed to the respective follow-up application. Here, we applied an optimized PP differentiation protocol to generate a GP2-enriched multipotent progenitor population suitable for subsequent endocrine, ductal, and acinar specification and highlighted the importance of the GP2^high^-expressing progenitor population. Using single-cell RNA sequencing analysis, we revealed relevant heterogeneity at the pancreatic progenitor stage and correlated the identified clusters with a human fetal dataset. Ligand-receptor analysis between the distinct clusters provided deeper insights into the complex interactions.

Powered by a comprehensive compound and growth factor testing setup, we determined the optimal media composition for generating high purity PPs. The results of the testing can serve other laboratories for further optimization or adjustment, considering potential cell line-dependent differences. True multipotency of the PPs was proven by simultaneous segregation into mature acinar, ductal, and endocrine structures upon orthotopic *in vivo* transplantation. However, based on our and other's previous efforts, transplantation of any PPs into an *in vivo* niche is likely to bypass limitations of *in vitro* protocols and usually properly generates all three lineages 7, 29, 44, 45.

As the newly generated PPs are particularly characterized by broad GP2 positivity, a recently identified multipotent progenitor marker 33-35, we further dissected the biology of GP2 expressing cells. We performed FACS-based GP2 sorting and challenged the subsequently purified GP2^high^, GP2^intermediate^ and GP2^negative^ PP population for their trilineage differentiation capacity.

The GP2^high^ population significantly outperformed the other two populations in terms of endocrine, acinar, and ductal differentiation. This indicates that not only GP2 positivity per se determines lineage potential, but also the GP2 expression level per cell. Therefore, we ascribe functional relevance to GP2 expressing PPs and define them as a trilineage competent population suitable for lineage engineering from the same ancestor into all three pancreatic lineages to closely resemble human fetal pancreatogenesis.

Further, the GP2-enriched PPs could be successfully applied for endocrine *in vitro* differentiation, generating functional stem cell-derived β-cells. They were also applicable for *in vitro* generation of PDLOs showing an improved ductal maturation state. Our new protocol in combination with a GP2 FACS purification step at the PP stage presents a progenitor population that can also be challenged for differentiation into PALOs. The ability to simultaneously generate endocrine, ductal, and acinar cells from the same progenitor population is a unique resource to decipher cell-type specific differences.

Using single cell-based transcriptomics of our newly generated PPs, we identified three progenitor clusters. Using GO term analysis and GSEA, we determined the identity as acinar-directed (progenytors 1), progenitor-like (progenitors 2) and ductal progenitor (progenitors 3). Hence, the entire pancreatic progenitor population therefore appeared to be heterogenous, harboring different cell types that are already primed for their respective cell lineage fates, exhibiting different degrees of maturity due to asynchronous differentiation 32.

In concordance with our *in vitro* data, we identified a GP2^high^ population at single-cell resolution correlating with acinar progenitor cells. GP2 was also moderately transcribed in the less specified pancreatic progenitor-like population, qualifying it as a valid PP marker in early pancreatic development. Another surface marker, F3 (=CD142), was also described for the enrichment of pancreatic progenitors 81 and correlated with GP2 expression in our new single cell dataset (**Figure S9A**). Since the higher specificity and applicability for GP2 over F3 has been described previously, we continued to focus on GP2 35. We further showed a strong correlation of the GP2^high^ population with fetal tip and trunk clusters at 7 to 10 wpc on single-cell transcriptome level. Thus, our single-cell resolved PP characterization landscape can instruct subsequent lineage differentiation protocols to generate more mature pancreatic cell types.

We further identified novel ligand-receptor interactions highlighting the complex signaling between the subpopulations, connected to cell lineage fate decisions at early pancreatic differentiation. Ligand-receptor signaling via midkine and NCL specifically connects the three progenitor clusters. The truncated isoform of midkine, a retinoic acid responsive gene, has already been linked to early organogenesis and detected at epithelial-mesenchymal interface 82. Its specific expression in highly proliferating cells also indicates a possible role in pancreas development 82. We could also validate the importance and functional role of midkine signaling *in vitro* by cluster-specific upregulation of pancreatic progenitor specific transcripts.

Putting these data into context of pancreatic lineage engineering, our systematic screening revealed unique details on the lineage determining effect of individual growth factors, leading to true multipotent PPs which have the potential to mature into the exocrine and endocrine compartment of the pancreas. The simultaneous generation of the different pancreatic lineages from a common precursor population will help us to decipher developmental cues and roots of human fetal pancreas development in very early stages. Particularly, high yields of GP2^high^ expressing PPs significantly boost the maturation grade of pancreatic ductal cells as evidenced by KRT positivity, now reaching cellular fractions of up to 30% of KRT7 of an entirely ductal lineage entered bulk. Furthermore, this provides the opportunity to investigate cell type-specific pathomechanisms of early cancer formation *in vitro* in organoid systems in a direct head-to-head comparison. With single-cell-based transcriptomics we demonstrated the heterogeneity and lineage fate commitment at the progenitor stage and identified important signaling patterns and cell-cell communications, a potential blueprint for future differentiation efforts.

## Methods

### Stem cell culture

Cultivation of hESCs and differentiation into the pancreatic lineage were approved by the Robert Koch Institute under the “79. Genehmigung nach dem Stammzellgesetz, AZ 3.04.02/0084”. The hESC line HUES8 was obtained from Harvard University (RRID: CVCL_B207) and H1 (University of Wisconsin/WiCell) cells were kindly provided by Maike Sander (Max-Delbrück-Center, Berlin). An in-house generated human iPSC line from a healthy donor and the WTC-11 iPSC line (cell line ID GM25256, Coriell Institute) 83, kindly received from Barbara Treutlein (ETH Zürich), were additionally used. Stem cells were cultured on hESC-qualified Matrigel (Corning)-coated plates in mTeSR1 or mTeSR Plus medium (STEMCELL Technologies) at 5% CO2, 5% O2, and 37 °C. Cells were passaged every 3 to 4 days at 80 - 100% confluency with TrypLE Express (Gibco) as previously described 23, 24.

### Design of the multi-compound testing strategy

Stage 1 compound testing was manually designed, whereas a multivariate experimental design was created using MODDE 12.1 software (Sartorius) for stage 2, stage 3, and stage 4 compound testing to minimize the number of experiments and maximize the output information. The various compounds were chosen based on published protocols 14-16, 21, 23, 24. Compound testing was performed on the differentiation protocol previously published by Breunig et al. 23, 24.

### Stage-specific compound testing for pancreatic differentiation

Pancreatic differentiation of hESC or iPSC lines was initiated in monolayer on growth factor reduced (GFR) Matrigel (Corning)-coated 24-well plates at 60 - 90% confluence as described previously 23, 24. The media composition was modified separately for each stage. The cytokines of the other stages and the basal media remained unchanged as recently described 23, 24. The medium was changed every 24 h. All experiments were performed in duplicates (two wells of one differentiation).

The detailed media compositions for stage 1, stage 2, stage 3, and stage 4 compound testing are listed in **Table S1, Table S2**, **Table S3,** and **Table S4**, respectively. Previously described basal media BE1 and BE3 were used in all approaches 23, 24. Media compositions of detailed testing of PKC activators (TPPB, Indolactam V) in stage 3 and stage 4 are shown in **Table S5**.

### Final Pancreatic differentiation medium composition

The final media composition was used for further PP differentiations and analysis: (i) Stage 1a: 1 day, BE1 + 0.1% BSA, 100 ng/mL Activin A, 2 µM CHIR99021 (CHIR), (ii) Stage 1b: 2 days, BE1 + 0.1% BSA, 100 ng/ml Activin A, 5 ng/mL FGF2, (iii) Stage 2: 3 days, BE1 + 0.5% BSA, 50 ng/mL FGF10, 3 ng/mL Wnt3A, 0.25 mM vitamin C, (iv) Stage 3: 3 days, BE3 + 2% BSA, 50 ng/mL noggin, 0.25 µM SANT-1, 1 µM retinoic acid, 16 mM glucose, 0.5 µM Indolactam V, (v) Stage 4: 4 days, BE3 + 2% BSA, 0.2 µM LDN-193189, 100 ng/mL EGF, 10 mM nicotinamide, 0.5 µM Indolactam V, 16 mM glucose, 0.25 µM vitamin C. Detailed information regarding the final protocol is listed in **Table S6**.

### Magnetic-activated cell sorting

At the pancreatic progenitor stage (stage 4) cells were harvested with TrypLE and blocked in 10% FCS in PBS for 30 min on ice. Cells were resuspended in anti-GP2 antibody (1:5000, MBL International) for 60 min on ice. After washing with 10% FCS in PBS, the cells were incubated with anti-mouse IgG micro beads (Miltenyi Biotec) for 15 min on ice. At this step, up to 10^7^ cells were resuspended in 80 µL 10% FCS in PBS and combined with 20 µL beads. After washing with FC buffer, the cells were incubated in donkey-anti-mouse Alexa Fluor 488 (1:500, Thermo Fisher) for 10 min on ice, followed by a washing step in PBS supplemented with 0.5% BSA and 2 mM EDTA (MACS buffer). Sorting was performed using MS columns in combination with a MiniMACS™ Separator (both Miltenyi Biotec). Columns were placed on the magnet and equilibrated with 500 µL MACS buffer. Cells were resuspended in 500 µL MACS buffer and loaded on the column. The column was washed three times with MACS buffer and the flow-through was collected. The column was removed from the magnet and cells were recovered with 500 µL MACS buffer using a plunger. Unstained, flow-through and GP2 positive sorted samples were used for further flow cytometry analysis.

### Fluorescence-activated cell sorting

Pancreatic progenitor cells were harvested on day 13 and blocked in FACS buffer (10% FCS, 1% penicillin-streptomycin (P/S), 10 µM Rock inhibitor Y-27632 in PBS) for 30 min on ice. The cells were stained with anti-GP2 primary antibody (1:5000) for 60 min on ice. Cells were washed with FACS buffer and incubated in donkey-anti-mouse Alexa Fluor 488 (1:500) secondary antibody for 10 min on ice. Cell sorting of GP2^negative^, GP2^intermediate^ and GP2^high^ populations was performed by the Core Facility Cytometry, Ulm University (FACSAria II, BD). After sorting, cells were further used for endocrine, ductal, or acinar differentiation as described below.

### Ductal differentiation

Cells were harvested at PP stage and further differentiated toward the ductal lineage as previously described with minor modifications 23, 24. Forskolin (10 µM; Sigma) was additionally added from day 20 on to increase KRT7 levels. Cells were harvested on day 27 or 28 for paraffin-embedding, flow cytometry analysis, and RNA isolation as previously outlined 23, 24.

### Endocrine differentiation

At PP stage, cells were further differentiated towards the endocrine lineage analogous to the protocol published by Hogrebe et al. 14. Subsequent to the PP stage, stage 5 medium was added, supplemented with Latrunculin for the first 24 h. The endocrine progenitor cells were harvested at stage 5 (day 20 or 21) or further differentiated into mature endocrine cells. Maturation was performed in enriched serum free medium (ESFM), either for 14 days in monolayer culture or for 7 days in monolayer culture, followed by suspension culture for another 7 days. Cells were harvested at each stage using TrypLE or fixed in PFA for downstream applications.

### Quantification of insulin secretion

For insulin secretion, PPs generated with the new protocol were further differentiated to β-cells in complete monolayer in a 24-well plate. At the end of stage 6, the cells were washed twice with KRBH buffer supplemented with 0.1% BSA. Cells were incubated in 300 µl KRBH containing 0.1% BSA and 1 mM glucose for 1 h at 37°C. The supernatant was removed and substituted with fresh KRBH supplemented with 0.1% BSA and 1 mM glucose. After 1 h incubation at 37°C, the supernatant was collected. Subsequently the cells were incubated in 300 µl KRBH containing 0.1% BSA and 20 mM glucose or 30 mM KCl for 1 h at 37°C. The supernatant was collected. An insulin ELISA Kit (ALPCO) was used to measure secreted insulin. The results were normalized to the total cell number per well.

### Acinar differentiation

HUES8 were differentiated to pancreatic progenitors using the improved protocol (**Table S6**) and PPs were sorted for GP2 expression by FACS as described above. The respective populations were subsequently used for the acinar differentiation. 35000 cells were seeded in a 50 µL matrigel dome in a 24-well plate. The matrigel domes were solidified at 37°C for 10 min and subsequently overlaid with acinar stage 5 medium. Acinar medium was changed every 4 days with stage 5, stage 6, stage 7, and stage 8 medium, respectively. Medium was composed as previously described 25. At day 28 (stage 8), acinar cells were harvested for histology or RNA extraction. To process organoids for RNA extraction, matrigel domes were washed with PBS and incubated with 500 µL 1 mg/mL collagenase/dispase solution at 37°C for 2 h. Cold neutralization solution (1% BSA, 1% P/S in DMEMF/12) was added to stop the enzymatic reaction. Cells were centrifuged and washed once with PBS. The cell pellet was directly used for RNA extraction as described below. For histology, matrigel domes were fixed in 4% PFA with 100 mM sucrose in PBS overnight at 4°C and further processed as described below.

### Orthotopic transplantation

All animal care and procedure were conducted in compliance with the German legal regulations and were previously approved by the local governmental review board of the state of Baden-Württemberg (Permission no. 1406). All mouse work aspects were performed according to acknowledged guidelines of the Society of Laboratory Animals (GV-SOLAS) and of the Federation of Laboratory Animal Science Associations (FELASA).

For orthotopic transplantation, cells were harvested at PP stage (day 13). Cells were seeded in stage 4 medium containing 10 µM Rock inhibitor Y-27632 on 6-well ultra-low attachment plates at a density of 6 x 10^5^ cells per ml medium. Spheres formed during a one-day incubation on an orbital shaker (95 rpm) at 37 °C. After 24 h, the spheres were washed twice with PBS. A subset of spheres was dissociated into single cells for counting and 1 Mio cells as whole spheres were resuspended in 25 µL stage 4 medium with 20 µM Y-27632 and 25 µL GFR-Matrigel. Transplantation into the murine pancreas was performed as described in detail previously 23, 24.

### RNA isolation, reverse transcription and quantitative real-time PCR

Cells were harvested as described above and RNA was isolated using the Gene-JET RNA Purification Kit (Thermo) according to the manufacturer's protocol as previously described 23, 24. cDNA was synthesized with the iScript cDNA Synthesis Kit (Bio-Rad). qPCR was performed with the GreenMasterMix (2x) No ROX (Genaxxon) on the Rotor-Gene Q (Qiagen). Expression levels were normalized to the housekeeping gene hydroxymethylbilane synthase (*HMBS*). The following primers were used: Hs_AFP_1_SG (QT00085183, Qiagen), Hs_AMY2A_2_SG (QT01680595, Qiagen), Hs_BHLHA15_1_SG (QT00201152, Qiagen), Hs_CA2_1_SG (QT00031059, Qiagen), Hs_CDH1_1_SG (QT00080143, Qiagen), Hs_CDH2_1_SG (QT00063196, Qiagen), Hs_CFTR_1_SG (QT00070007, Qiagen), Hs_CPA1_1_SG (QT00001736, Qiagen), Hs_CTSB_1_SG (QT00088641, Qiagen), Hs_DSC2_1_SG (QT00016128, Qiagen), Hs_FOXA1_1_SG (QT00212828, Qiagen), Hs_GATA4_1_SG (QT00031997, Qiagen), Hs_GCG_1_SG (QT00091756, Qiagen), Hs_GLIS3_1_SG (QT00037702, Qiagen),Hs_GP2_1_SG (QT00010535, Qiagen), Hs_HMBS_1_SG (QT00014462, Qiagen), Hs_INS_2_SG (QT01531040, Qiagen), Hs_MUC1_2_SG (QT01667239, Qiagen), Hs_NKX2-2_SG (QT00200158, Qiagen), Hs_NKX6-1_1_SG (QT00092379, Qiagen), Hs_PROX1_1_SG (QT01006670, Qiagen), Hs_PTF1A_2_SG (QT01033396, Qiagen), Hs_SOX17_1_SG (QT00204099, Qiagen), Hs_SOX2_1_SG (QT00237601, Qiagen), Hs_SOX9_1_SG (QT00001498, Qiagen), KRT19 (CTACAGCCACCACGAC, CAGAGCCTGTCTCAAA, Biomers), KRT7 (ATAGCCAGCTGTCCCGAATG, GCCTGGAGAAGTCATTGCT, Biomers), vimentin (GACAATGCGTCTCTGGCACGTCTT, TCCTCCGCCTCCTGCAGGTTCTT, Biomers), XBP1 (TGCTGAGTCCCAGGTG, GTCGGCAGGCGGGAAG, Biomers).

### Compound treatment of pancreatic progenitors

For the *in vitro* validation of *in silico* predicted ligand-receptor interactions, pancreatic progenitors were treated either with 10 ng/mL recombinant human midkine or 50 ng/mL recombinant human VEGF (both Peprotech), resuspended in stage 4 differentiation medium. The cells were harvested after 30 h of treatment for subsequent RNA extraction and mRNA expression analysis. An untreated control was used for comparison.

### Flow cytometry

Staining of the surface markers CD56 (anti CD56 AF488-conjugated, 1:50, BD), GP2 (anti-GP2, 1:5,000), CXCR4 (anti-CXCR4 PE-conjugated, 1:50, Life Technologies), C-KIT (anti-C-KIT APC-conjugated, 1:100, Thermo), TRA1-60 (anti-TRA1-50 FITC-conjugated, 1:10, BD), and SSEA4 (anti-SSEA4 PE-conjugated, 1:10, BD) was performed as previously described 23, 24.

For intracellular staining, cells were washed with PBS after harvesting and fixed in 4% paraformaldehyde (PFA) and 100 mM sucrose (both Sigma) in PBS for 25 min on ice. The intracellular stainings were performed as recently described in Breunig et al. In brief, the PFA-fixed cells were blocked and permeabilized in 5% normal donkey serum (NDS, Jackson ImmunoResearch), 0.1% Triton X-100 in PBS for 30 min on ice. The primary antibodies were added overnight at 4 °C. The secondary antibodies donkey-anti-rabbit, donkey-anti-mouse, and donkey-anti-goat (Alexa Fluor 488-, 568-, 647-conjugated, 1:500, Thermo Fisher) were added for 90 min on ice. The following antibodies were used: anti-NKX6-1 (1:150, DSHB), anti-PDX1 (1:500, R&D), anti-CDX2 (1:500, Cell Marque), anti-AFP (1:200, Dako), anti-SOX17 (1:500, R&D), SOX2 (1:300, R&D), anti-GATA4-PE (1:50, BD), anti-vimentin (1:100, Cell Signaling), anti-CPEP (1:200, Cell Signaling), anti-GCG (1:1000, Sigma), anti-KRT7 (1:200 Dako).

Flow cytometry measurement was either performed with the LSRII (BD) or with Attune^TM^ NxT (ThermoFisher) flow cytometer. The flow cytometry results were analyzed using FlowJo (V10.7.1).

### Multivariate linear regression analysis

For the compound testing, the expression of various marker genes (NKX6-1, PDX1, GP2, GATA4, CD56, VIM, SOX2, SOX17, AFP, CDX2) was monitored at PP stage with FC. Multivariate linear regression analysis was performed on FC results with JASP statistical software (V0.14, JASP Team).

The flow cytometry results for each marker were set as a dependent variable. The tested compounds and respective concentrations were set as independent variables. The standardized regression coefficients (β) of the multivariate linear regression analysis are demonstrating the positive (increasing) or negative (decreasing) effect of the independent variable on the dependent variable. In addition, a p-value indicates whether this relationship is significant (p < 0.05: *, p < 0.01: **, p < 0.001: ***). The normalized regression coefficients of the multivariate linear regression analysis are depicted as a heatmap.

### Immunocytochemistry staining

For in-well immunofluorescence stainings, pancreatic differentiation was performed on GFR Matrigel-coated ibiTreat µ-plates (ibidi). At PP stage, cells were washed twice with PBS and fixed with 4% PFA/ 100 mM sucrose/ PBS for 30 min at room temperature. Cells were washed with PBS. For intracellular marker staining, cells were blocked and permeabilized in 5% NDS/ 0.1% Triton X-100/ PBS for 30 min at room temperature. For surface staining, cells were blocked in 10% FCS/ PBS for 30 min at room temperature. Afterwards, primary antibody was added overnight at 4°C for the intracellular markers CPA1 (1:200, BioRad), CPEP (1:200, cell signaling), and GCG (1:1000, Sigma) and 90 min at room temperature for the surface marker GP2 (1:1000, MBL international). Cells were washed and subsequently incubated with the secondary antibody for 90 min (intracellular marker) or 15 min for surface markers. Counterstaining was performed with 500 ng/mL DAPI. After final washing steps, wells were covered by PBS and imaged with a Zeiss Axioscope2 microscope (Carl Zeiss).

### Histology

Murine pancreata were fixed in 4% formaldehyde overnight. Organoids were fixed in 4% PFA in PBS with 100 mM sucrose overnight. After a standard automated dehydration series tissues were embedded in paraffin. 4 µm thick sections were prepared. H&E staining and immunofluorescence stainings were performed as described previously 23, 24. The following primary antibodies were used for immunostaining: acTUB (1:1000, Abcam), AFP (1:500, DAKO), AMY1A (1:100, Merck), ARL13B (1:1000, Abcam), CDX2 (1:500, Cell Marque), CFTR (1:200, R&D), CHGA (1:200, DAKO), CPA1 (1:1000, BioRad), CTRC (1:500, Millipore), Ecad (1:200, cell signaling), FOXA2 (1:500, Abcam), GCG (1:1000, Sigma), GP2 (1:100, MBL international), INS (1:5000, Abcam), KRT19 (1:100, DAKO), KRT7 (1:200), NKX6-1 (1:150, DSHB), PDX1 (1:500, R&D), SOX2 (1:300, R&D), SOX9 (1:500, Millipore), TRY (1:50, Santa Cruz), VIM (1:500, Cell Signaling). Donkey Alexa Fluor secondary antibodies (1:500, Thermo Fisher) were used. Images were taken with a Zeiss Axioscope2 microscope combined with a Plan Apo 20x/0.8 objective (Carl Zeiss). Brightness and contrast of the images were only adjusted to improve illustration with ImageJ software (V1.53c, National Institute of Health). Images were quantified using CellProfiler (V4.2.4, Broad Institute) 84. Either the number or area of detected structures was quantified and normalized to DAPI positive cells.

### Statistical analysis

Statistical analyses were conducted with GraphPad Prism (GraphPad). Two groups were compared with two-tailed t-test. One-way ANOVA tests were performed for qPCR and flow cytometry data comparing at least 3 groups. Bar graphs show the mean ± SEM and significance is indicated as p < 0.05: *, p < 0.01: **, p < 0.001: ***, p < 0.0001: ****. Statistical analysis was performed for experiments with n=3 independent differentiations. Indicated replicates refer to independent wells of one differentiation.

### Harvesting and processing PPs for single-cell RNA sequencing

For the scRNA-seq experiment, the PPs were harvested and processed on day 13 of the differentiation. Control FC stainings showed a percentage of 86% PDX1 and NKX6-1 double-positive cells. Furthermore, 89% of the cells were positive for GP2 (data not shown). First, the cells were washed with 1 mL PBS (Gibco) per well. Afterward, 250 µL TrypLE Select was added for 8 min to detach and individualize the cells. 750 µL of DMEM/F12 (Gibco) stopped the reaction. The 2.1 million PPs with a viability of 89% were centrifuged at 300 rpm for 5 min and resuspended in 1 mL DMEM/F12. The PPs were loaded with a target cell number of 10,000 cells. Applying the manufacturer's protocol, the Chromium Single Cell 3′ Kit v3.1 (10x Genomics) was used to generate the RNA and cDNA library. The cDNA library was sequenced on an S1 flow cell (Illumina) in XP mode.

### Processing of available single-cell RNA sequencing datasets

Pancreatic progenitor sample of Hogrebe et al. 36 was obtained under the accession number GSM4083856 in the Gene Expression Omnibus. Data processing was performed using SeuratV4.1.1 85. The matrix was filtered for cells with at least 200 genes and gene expression in minimum 3 cells. Next, duplicate cells, cells with more than 15% mitochondrial genes and cells with less than 1,200 genes were filtered out. Data were normalized with NormalizeData function. Mitochondrial genes were regressed out using ScaleData function. UMAP was calculated using 17 PCAs and a resolution of 0.4.

### scRNA-seq processing

The raw sequencing fastq files were further processed with CellRanger (10x Genomics). They were demultiplexed, aligned to the human reference genome (GRCh38 2020-A), and filtered. The unique molecular identifiers (UMIs) and barcodes were counted and quality filtered. If not stated differently, the following downstream analyses were performed with Scanpy API 54 with default settings. The filtered count matrix of the 5,027 cells was loaded and first filtered for cells with a minimum of 200 genes and genes expressed in at least three cells. In the next step, cells with less than 1,200 genes and more than 15% mitochondrial genes were filtered out. The remaining 4,236 cells had an average of 5,813 genes and 31,903 total counts per cell. Furthermore, mitochondrial, and ribosomal genes were removed. The counts were normalized to 10,000 counts per cell and logarithmized. 2,156 highly variable genes were found, and total counts, percentage of mitochondrial genes, ribosomal genes, and cell cycle were regressed out. The data were scaled to unit variance, were used together with ten nearest neighbors to calculate the neighborhood graph. The data were clustered with the Leiden algorithm with a resolution of 0.4 into seven cell clusters. The UMAP was calculated with init_pos='paga'. The top 300 characterizing genes (Table S7) per Leiden cluster were calculated with the t-test method. The cell clusters were annotated according to known marker genes. The day 13 PP sample from Wiedenmann, Breunig 54 (GSE162547) was processed the same, except that only 24 PCAs and a resolution of 0.3 had been used in the Leiden algorithm. The quality filtering reduced the cell number from 7,893 to 6,732 cells. 1,814 highly variable genes were found. The top 300 characterizing genes can be found in **Table S7.**

For the correlation plot (**Figure 8B**), the intersection of the highly variable genes (1,171 genes) of both datasets was used. The mean expression value of each gene per cluster and dataset was calculated. Then the pairwise correlation of the clusters was calculated with the Pearson correlation coefficient.The versions of applied softwares are python 3.9.5, jupyterlab 3.3.1, IPython 8.1.1, 10x Genomics Cell Ranger 6.1.2, anndata 0.8.0, scanpy 1.8.2, leidenalg 0.8.9, matplotlib 3.5.1, numpy 1.21.5, pandas 1.4.1, scipy 1.8.0, seaborn 0.11.2, sklearn 1.0.2, xlsxwriter 3.0.3.

Unsupervised hierarchical clustering combining marker genes and cluster memberships of the in-house and published fetal dataset was performed using ClusterMap 86 in the R computational platform 87. Briefly, we first obtained UMAP cluster membership and corresponding maker genes of the published dataset 60. We merged this with our single-cell dataset corresponding information. ClusterMap was then used to generate unsupervised regrouping of cell memberships between the two datasets based on a similarity metrics. The results were displayed either using heatmaps 88 or dendrograms.

### Ligand-receptor analysis

The cell-cell communication was undertaken using CellChat 70. We first normalized and log transformed raw single-cell count data using Seurat 89. The CellChat object was then created using the normalized data and UMAP cluster membership based on our previous analysis. Communication probability was then computed and filtered to exclude clusters with lower than 10 cells. Signaling pathways showing significant communications (p-value < 0.05) were used for downstream in-depth analysis including network aggregation, ligand-receptor pairs, cell-cell communication network, assessment of outgoing and incoming signals between clusters, and visual embedding based on either functional or structural similarities of signaling pathways.

## Supplementary Material

Supplementary figures and tables 1-5.

Supplementary table 6.

Supplementary table 7.

Supplementary table 8.

Supplementary table 9.

## Abbreviations

acTUB: acetylated tubulin
BMP: bone morphogenic protein
CA2: carbonic anhydrase
CHGA: chromogranin A
CHIR: CHIR99021
CPA1: carboxypeptidase 1
CPEP: c-peptide
CTRC: chymotrypsin C
DE: definitive endoderm
ESFM: enriched serum free medium
FACS: fluorescence activated cell sorting
FAF-BSA: fatty-acid-free bovine serum albumin
FC: flow cytometry
GCG: glucagon
GFR: growth-factor reduced
GO: gene ontology
GP2: glycoprotein 2
GSEA: gene set enrichment analysis
GSIS: glucose-stimulated insulin secretion
GTE: gut tube endoderm
H&E: hematoxylin and eosin
hESC: human embryonic stem cell
HMBS: hydroxymethylbilane synthase
hPSC: human pluripotent stem cell
ILV: Indolactam V
INS: insulin
iPSC: induced pluripotent stem cell
ITS-X: insulin-transferrin-selenium-ethanolamine
KRT19: keratin 19
KRT7: keratin 7
LDN: LDN-193189
L-R: ligand-receptor
MACS: magnetic-activated cell sorting
MDK: midkine
MUC1: mucin 1
NCL: nucleolin
NDS: normal donkey serum
NES: normalized enrichment score
NSG: NOD *scid* gamma mice
P/S: penicillin-streptomycin
PALO: pancreatic acinar-like organoid
PDLO: pancreatic duct-like organoid
PE: pancreatic endoderm
PFA: paraformaldehyde
PKC: protein kinase C
PP: pancreatic progenitor
PTN: pleiotrophin
scRNA-seq: single-cell RNA sequencing
TRY: trypsin
UMAP: Uniform Manifold Approximation and Projection
VIM: vimentin
wpc: weeks post-conception
