# Supplementary material for: Single-cell profiling of GP2-enriched pancreatic progenitors to simultaneously create acinar, ductal, and endocrine organoids
Source: Theranostics. 2023 Mar 21;13(6):1949–73. doi: 10.7150/thno.78323 (PMC10091881; doi:10.7150/thno.78323)
Supplement: Supplementary file 1 — Supplementary figures and tables 1-5. [file thnov13p1949s1.pdf]

Figure S1

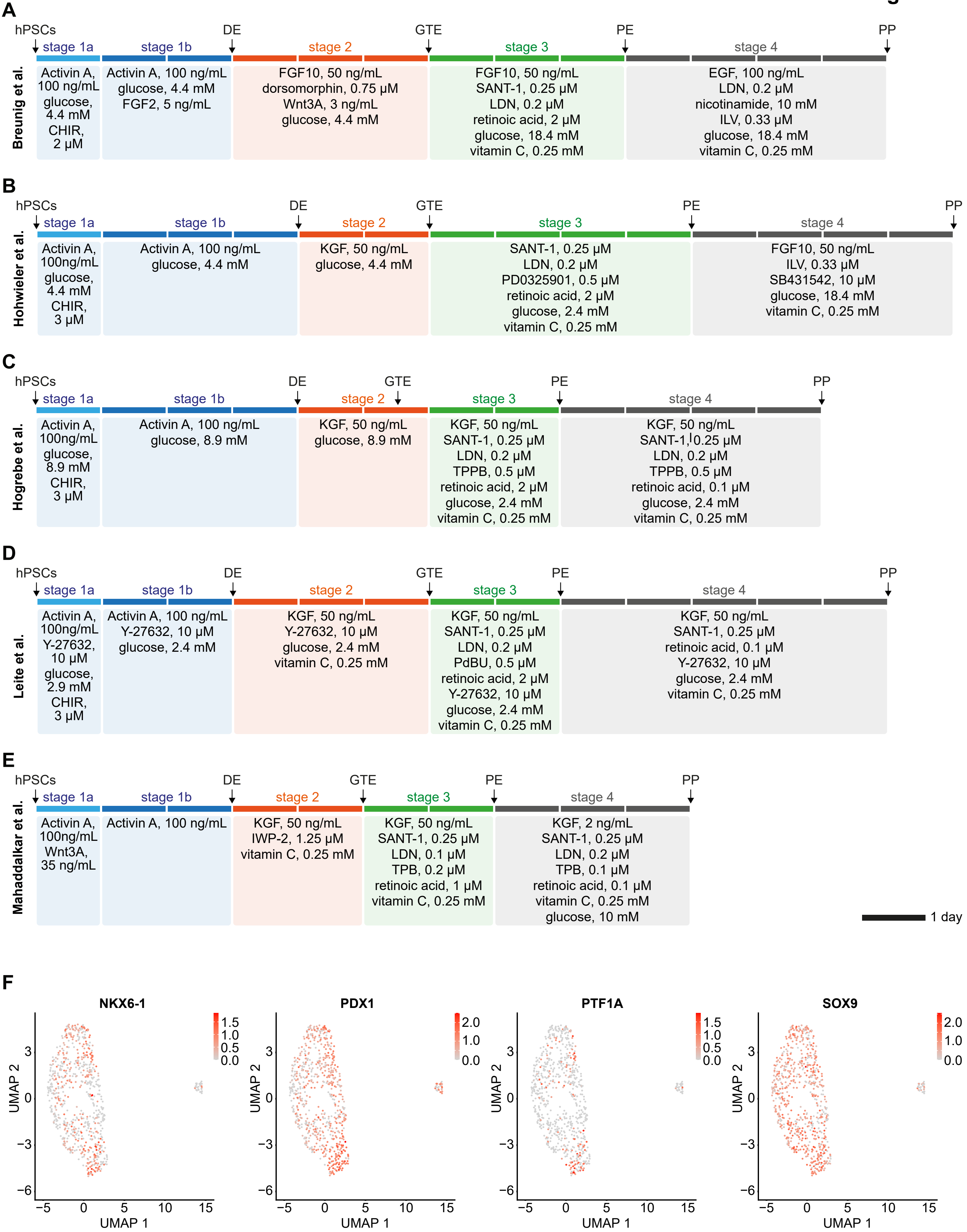
A
Breunig et al.
hPSCs
DE
GTE
PE
PP
stage 1a
stage 1b
stage 2
stage 3
stage 4
Activin A, 100 ng/mL glucose, 4.4 mM CHIR, 2 μM
Activin A, 100 ng/mL glucose, 4.4 mM FGF2, 5 ng/mL
FGF10, 50 ng/mL dorsomorphin, 0.75 μM Wnt3A, 3 ng/mL glucose, 4.4 mM
FGF10, 50 ng/mL SANT-1, 0.25 μM LDN, 0.2 μM retinoic acid, 2 μM glucose, 18.4 mM vitamin C, 0.25 mM
EGF, 100 ng/mL LDN, 0.2 μM nicotinamide, 10 mM ILV, 0.33 μM glucose, 18.4 mM vitamin C, 0.25 mM
B
Hohwieler et al.
hPSCs
DE
GTE
PE
PP
stage 1a
stage 1b
stage 2
stage 3
stage 4
Activin A, 100ng/mL glucose, 4.4 mM CHIR, 3 μM
Activin A, 100 ng/mL glucose, 4.4 mM
KGF, 50 ng/mL glucose, 4.4 mM
SANT-1, 0.25 μM LDN, 0.2 μM PD0325901, 0.5 μM retinoic acid, 2 μM glucose, 2.4 mM vitamin C, 0.25 mM
FGF10, 50 ng/mL ILV, 0.33 μM SB431542, 10 μM glucose, 18.4 mM vitamin C, 0.25 mM
C
Hogrebe et al.
hPSCs
DE
GTE
PE
PP
stage 1a
stage 1b
stage 2
stage 3
stage 4
Activin A, 100ng/mL glucose, 8.9 mM CHIR, 3 μM
Activin A, 100 ng/mL glucose, 8.9 mM
KGF, 50 ng/mL glucose, 8.9 mM
KGF, 50 ng/mL SANT-1, 0.25 μM LDN, 0.2 μM TPPB, 0.5 μM retinoic acid, 2 μM glucose, 2.4 mM vitamin C, 0.25 mM
KGF, 50 ng/mL SANT-1, 0.25 μM LDN, 0.2 μM TPPB, 0.5 μM retinoic acid, 0.1 μM glucose, 2.4 mM vitamin C, 0.25 mM
D
Leite et al.
hPSCs
DE
GTE
PE
PP
stage 1a
stage 1b
stage 2
stage 3
stage 4
Activin A, 100ng/mL Y-27632, 10 μM glucose, 2.9 mM CHIR, 3 μM
Activin A, 100 ng/mL Y-27632, 10 μM glucose, 2.4 mM
KGF, 50 ng/mL Y-27632, 10 μM glucose, 2.4 mM vitamin C, 0.25 mM
KGF, 50 ng/mL SANT-1, 0.25 μM LDN, 0.2 μM PdBU, 0.5 μM retinoic acid, 2 μM Y-27632, 10 μM glucose, 2.4 mM vitamin C, 0.25 mM
KGF, 50 ng/mL SANT-1, 0.25 μM retinoic acid, 0.1 μM Y-27632, 10 μM glucose, 2.4 mM vitamin C, 0.25 mM
E
Mahaddalkar et al.
hPSCs
DE
GTE
PE
PP
stage 1a
stage 1b
stage 2
stage 3
stage 4
Activin A, 100ng/mL Wnt3A, 35 ng/mL
Activin A, 100 ng/mL
KGF, 50 ng/mL IWP-2, 1.25 μM vitamin C, 0.25 mM
KGF, 50 ng/mL SANT-1, 0.25 μM LDN, 0.1 μM TPB, 0.2 μM retinoic acid, 1 μM vitamin C, 0.25 mM
KGF, 2 ng/mL SANT-1, 0.25 μM LDN, 0.2 μM TPB, 0.1 μM retinoic acid, 0.1 μM vitamin C, 0.25 mM glucose, 10 mM
1 day
F
NKX6-1
PDX1
PTF1A
SOX9
UMAP 1
UMAP 2
−5 0 5 10 15
−6 −3 0 3
0.0 0.5 1.0 1.5
0.0 1.0 2.0

Figure S1. Overview of established protocols for the four-step PP generation with DE, GTE and PE as intermediate stages. Protocols from (**A**) Breunig et al. [1, 2], (**B**) Hohwieler et al. [3], (**C**) Hogrebe et al. [4], (**D**) Leite et al. [5] and (**E**) Mahaddalkar et al. [6] are depicted. (**F**) UMAPs showing the expression of *NKX6-1*, *PDX1*, *PTF1A*, and *SOX9* across the 5 clusters of a published single cell RNA sequencing data set (Hogrebe et al. [7]). CHIR: CHIR99021; DE: definitive endoderm; GTE: gut tube endoderm; ILV: Indolactam V; PE: pancreatic endoderm; PP: pancreatic progenitor

Figure S2

A

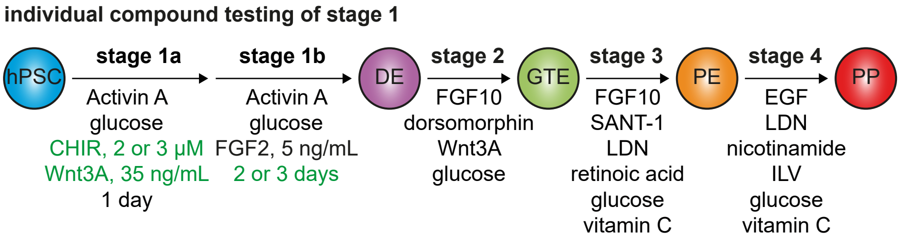

B

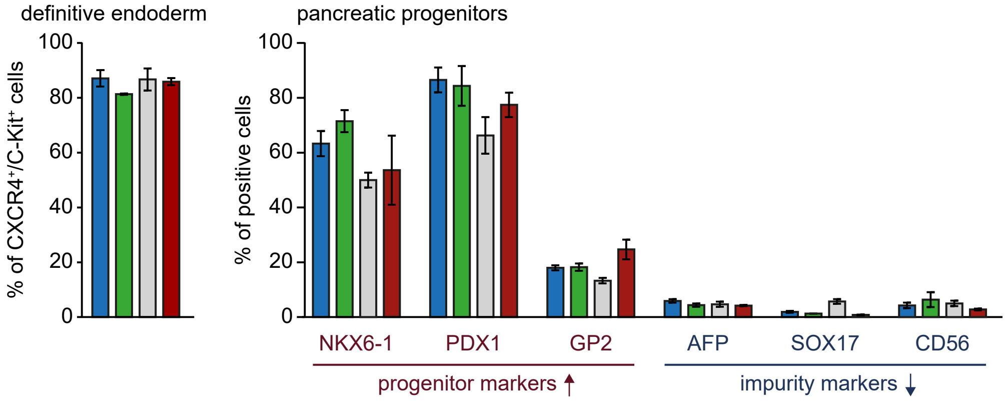

	stage 1a				stage 1b			
blue	*Activin A 100 ng/mL*	*glucose 4.4 mM*	CHIR 2 μM	1 day	*Activin A 100 ng/mL*	*glucose 4.4 mM*	*FGF2 5 ng/mL*	2 days
green	*Activin A 100 ng/mL*	*glucose 4.4 mM*	CHIR 3 μM	1 day	*Activin A 100 ng/mL*	*glucose 4.4 mM*	*FGF2 5 ng/mL*	2 days
grey	*Activin A 100 ng/mL*	*glucose 4.4 mM*	Wnt3A 35 ng/mL	1 day	*Activin A 100 ng/mL*	*glucose 4.4 mM*	*FGF2 5 ng/mL*	2 days
red	*Activin A 100 ng/mL*	*glucose 4.4 mM*	CHIR 2 μM	1 day	*Activin A 100 ng/mL*	*glucose 4.4 mM*	*FGF2 5 ng/mL*	3 days

Figure S2. Stage 1 compound testing reveals minor influence on PP generation. (**A**) Schematic overview of the individual compound testing for optimization of stage 1. The tested compounds are highlighted in red. (**B**) Flow cytometry results of CXCR4/C-KIT staining at definitive endoderm (DE) and of progenitors (NKX6-1, PDX1, GP2) and impurity markers (AFP, SOX17, CD56) at the pancreatic progenitor (PP) stage. The condition highlighted in red was used in the final protocol. Compounds that are written *in italics* were not varied. Mean, n=3. CHIR: CHIR99021; GP2: glycoprotein 2; GTE: gut tube endoderm; ILV: Indolactam V; LDN: LDN-193189; PE: pancreatic endoderm; VIM: vimentin

Figure S3

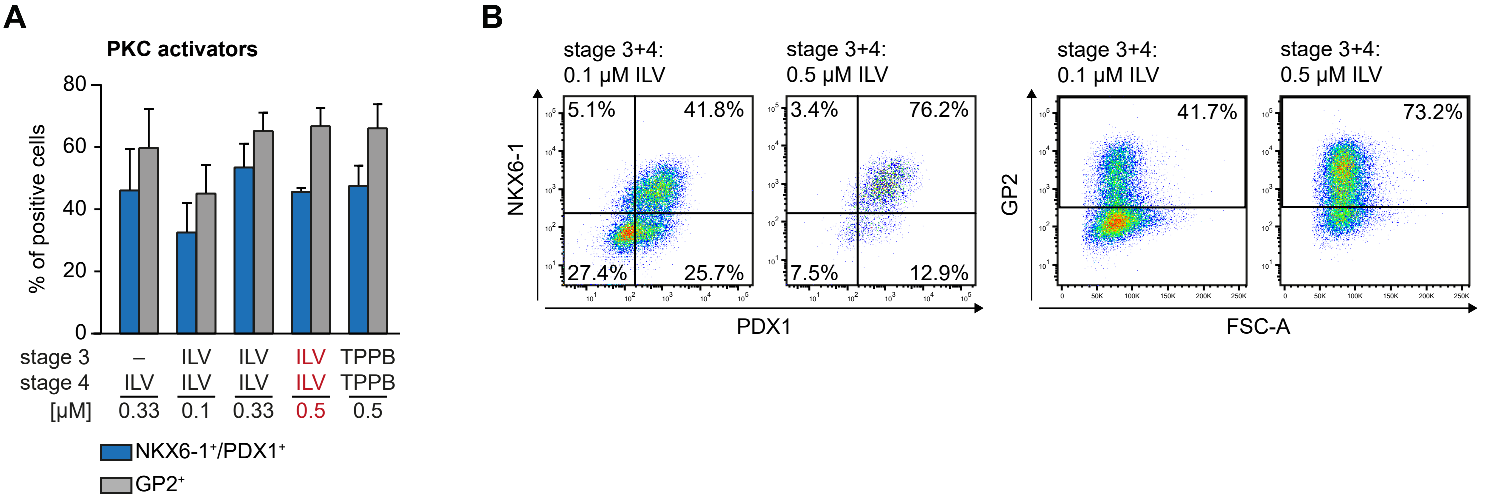

A
PKC activators
% of positive cells
80
60
40
20
0
stage 3
stage 4
[µM]
–
ILV
0.33
ILV
ILV
0.1
ILV
ILV
0.33
ILV
ILV
0.5
TPPB
TPPB
0.5
NKX6-1+/PDX1+
GP2+
B
stage 3+4: 0.1 µM ILV
stage 3+4: 0.5 µM ILV
NKX6-1
PDX1
5.1%
41.8%
27.4%
25.7%
3.4%
76.2%
7.5%
12.9%
GP2
FSC-A
41.7%
73.2%

Figure S3. Influence of PKC activation on pancreatic progenitor generation. (**A**) Different concentrations and combinations of the PKC activators ILV (0.1 µM, 0.33 µM, 0.5 µM) and TPPB (0.5 µM) were analyzed in a separate experiment. Flow cytometry results of NKX6-1/PDX1 double positive population and GP2 positive cells are depicted. Mean ± SEM, n=3. Significance was evaluated with two-way ANOVA, $P < 0.05$: *, $p < 0.01$: **, $p < 0.001$: ***. (**B**) Representative flow cytometry plots of NKX6-1/PDX1 and GP2 staining for various combinations and concentrations of PKC activators TPPB and ILV. GP2: glycoprotein 2; ILV: Indolactam V; PKC: protein kinase C

Figure S4

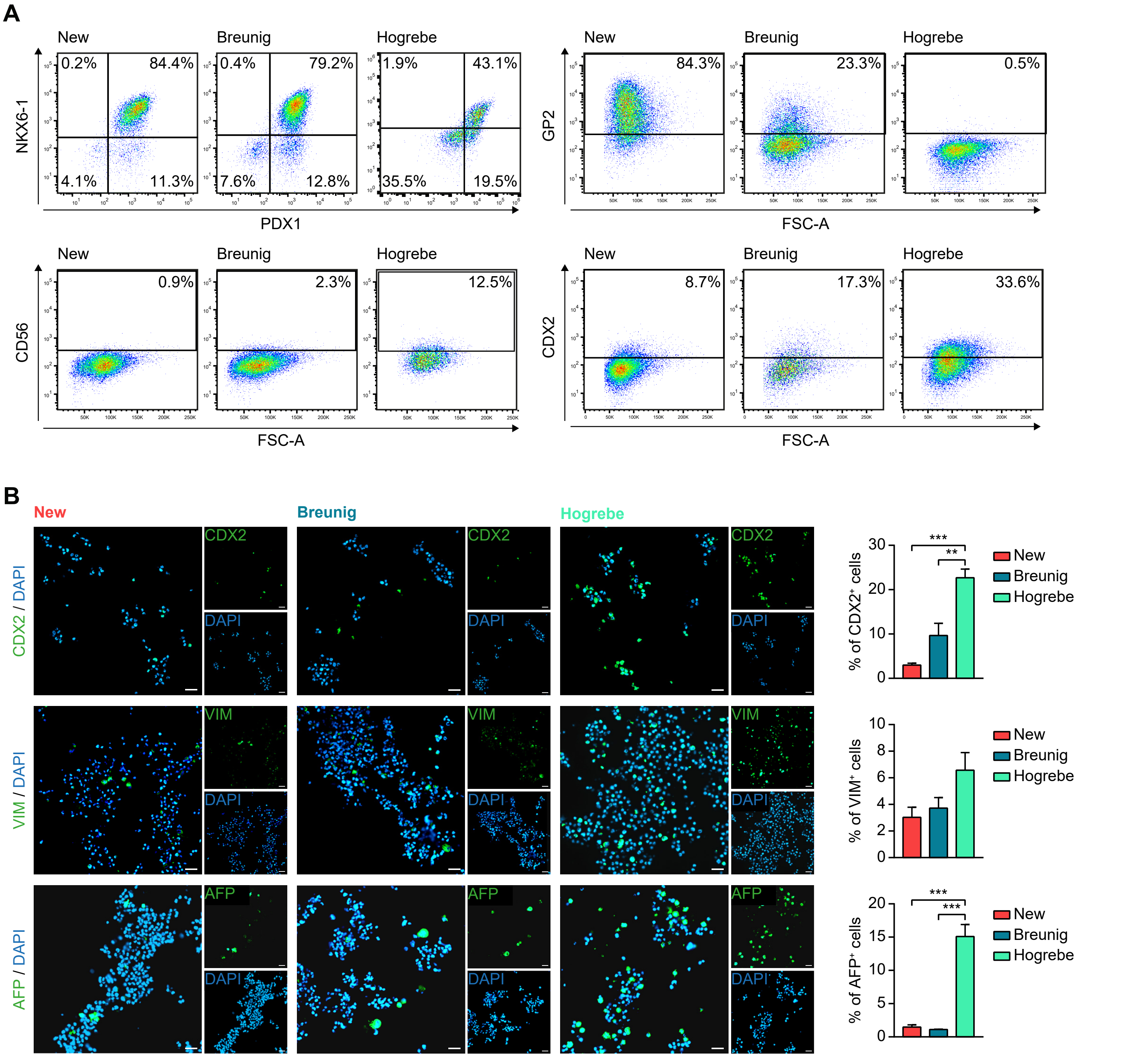
A
New
Breunig
Hogrebe
NKX6-1
PDX1
0.2%
84.4%
4.1%
11.3%
0.4%
79.2%
7.6%
12.8%
1.9%
43.1%
35.5%
19.5%
GP2
FSC-A
84.3%
23.3%
0.5%
CD56
0.9%
2.3%
12.5%
CDX2
8.7%
17.3%
33.6%
B
CDX2 / DAPI
VIM / DAPI
AFP / DAPI
CDX2
DAPI
VIM
AFP
% of CDX2⁺ cells
% of VIM⁺ cells
% of AFP⁺ cells
***
**

Figure S4. Characterization of pancreatic progenitors. (**A**) Representative flow cytometry plots of NKX6-1/PDX1, GP2, CD56 and CDX2 staining of PPs generated with the new, Breunig [1, 2] and Hogrebe [4] protocol. (**B**) Representative immunofluorescence stainings of the impurity markers CDX2 (green), VIM (green), and AFP (green) are shown along with their quantifications. CDX2 (n=4 fields of view), VIM (n=3 fields of view) and AFP (new n=3, Breunig n=3, Hogrebe n=4 fields of view) positive cells were normalized to DAPI. Scale bars represent 50 µm. Significance was assessed with an ordinary one-way ANOVA, $P < 0.05$: *, $p < 0.01$: **, $p < 0.001$: ***. GP2: glycoprotein 2; PP: pancreatic progenitor; VIM: vimentin

Figure S5

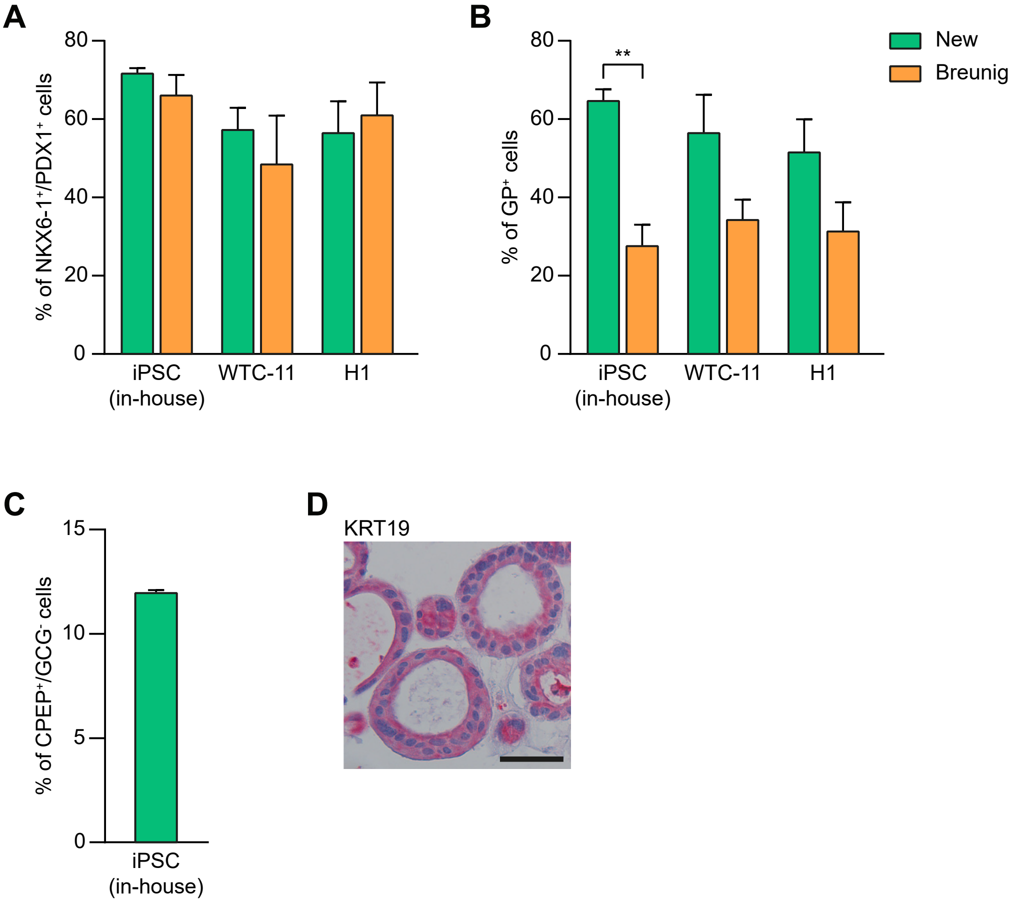

A
80
60
40
20
0
% of NKX6-1⁺/PDX1⁺ cells
iPSC (in-house)
WTC-11
H1
B
**
% of GP⁺ cells
New
Breunig
C
15
10
5
% of CPEP⁺/GCG⁻ cells
D
KRT19

Figure S5. Elevated GP2 expression upon PP generation with the new protocol is reproducible across various cell lines. An in-house generated iPSC line, WTC iPSC line and H1 hESC line were differentiated to pancreatic progenitors using the new or Breunig protocol [1, 2]. (**A**) FC analysis results of NKX6-1 and PDX1 double positive population and (**B**) GP2 positive cells are shown**.** Bars represent mean ± SEM, n=6 (in-house iPSC, H1) or n=4 (WTC). Significance was determined with two-tailed t-test, $P < 0.05$: *, $p < 0.01$: **, $p < 0.001$: ***. (**C**) Bar graph shows the $CPEP^+/GCG^-$ cells (flow cytometry) of endocrine progenitors generated by applying the new protocol to an in-house generated iPSC line in technical duplicates. (**D**) Ductal differentiation with the new protocol of an in-house generated iPSC line was shown with IHC staining of KRT19. Scale bars represent 50 µm. GP2: glycoprotein 2; iPSC: induced pluripotent stem cell

Figure S6

A

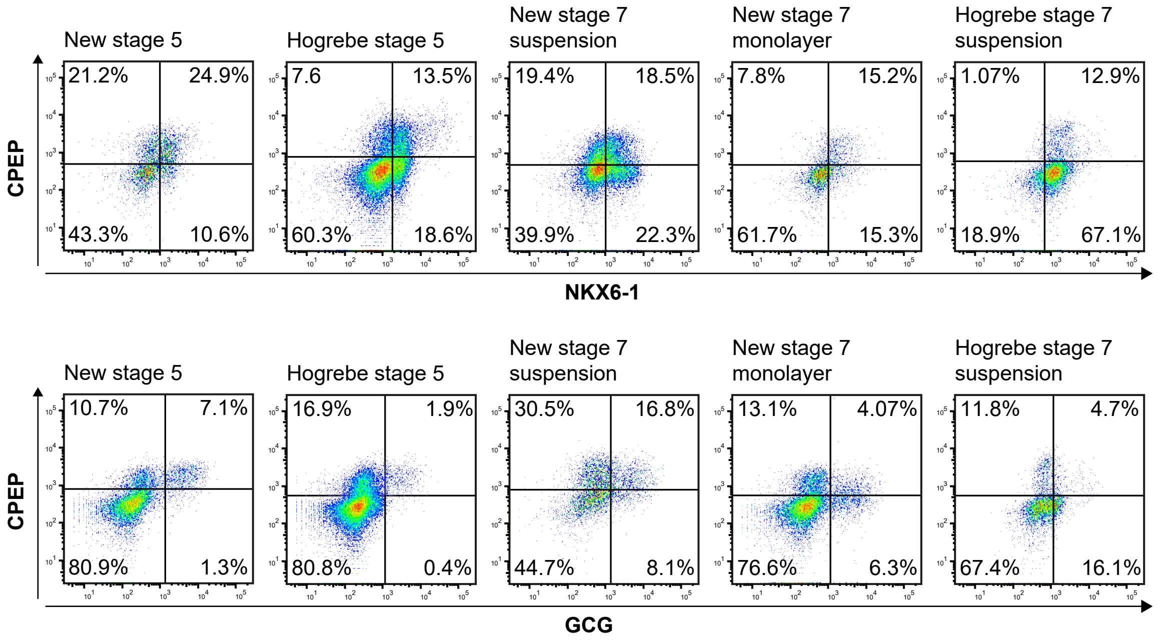
New stage 5
Hogrebe stage 5
New stage 7 suspension
New stage 7 monolayer
Hogrebe stage 7 suspension
CPEP
NKX6-1
21.2%
24.9%
43.3%
10.6%
7.6
13.5%
60.3%
18.6%
19.4%
18.5%
39.9%
22.3%
7.8%
15.2%
61.7%
15.3%
1.07%
12.9%
18.9%
67.1%
GCG
10.7%
7.1%
80.9%
1.3%
16.9%
1.9%
80.8%
0.4%
30.5%
16.8%
44.7%
8.1%
13.1%
4.07%
76.6%
6.3%
11.8%
4.7%
67.4%
16.1%

B

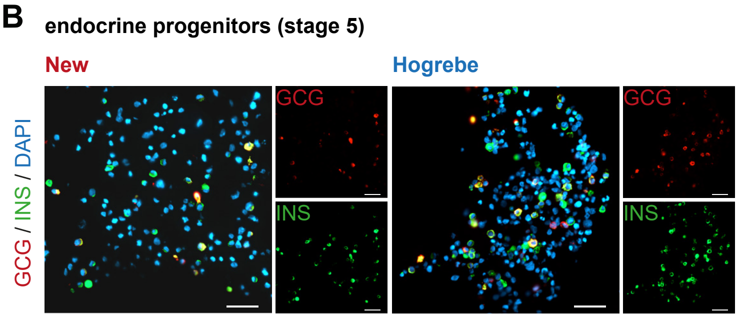
endocrine progenitors (stage 5)
New
Hogrebe
GCG / INS / DAPI
GCG
INS

Figure S6. Characterization of stem-cell derived endocrine cells. (**A**) Representative FC plots (NKX6-1/CPEP, GCG/CPEP) at the endocrine stage 5 and stage 6 of cells generated with the new or Hogrebe [4] protocol are shown. (**B**) Immunofluorescence staining for GCG (red) and INS (green) of endocrine progenitors (stage 5). Counterstaining was performed with DAPI (blue). Scale bars represent 100 µm. CPEP: c-peptide; GCG: glucagon; INS: insulin

Figure S7

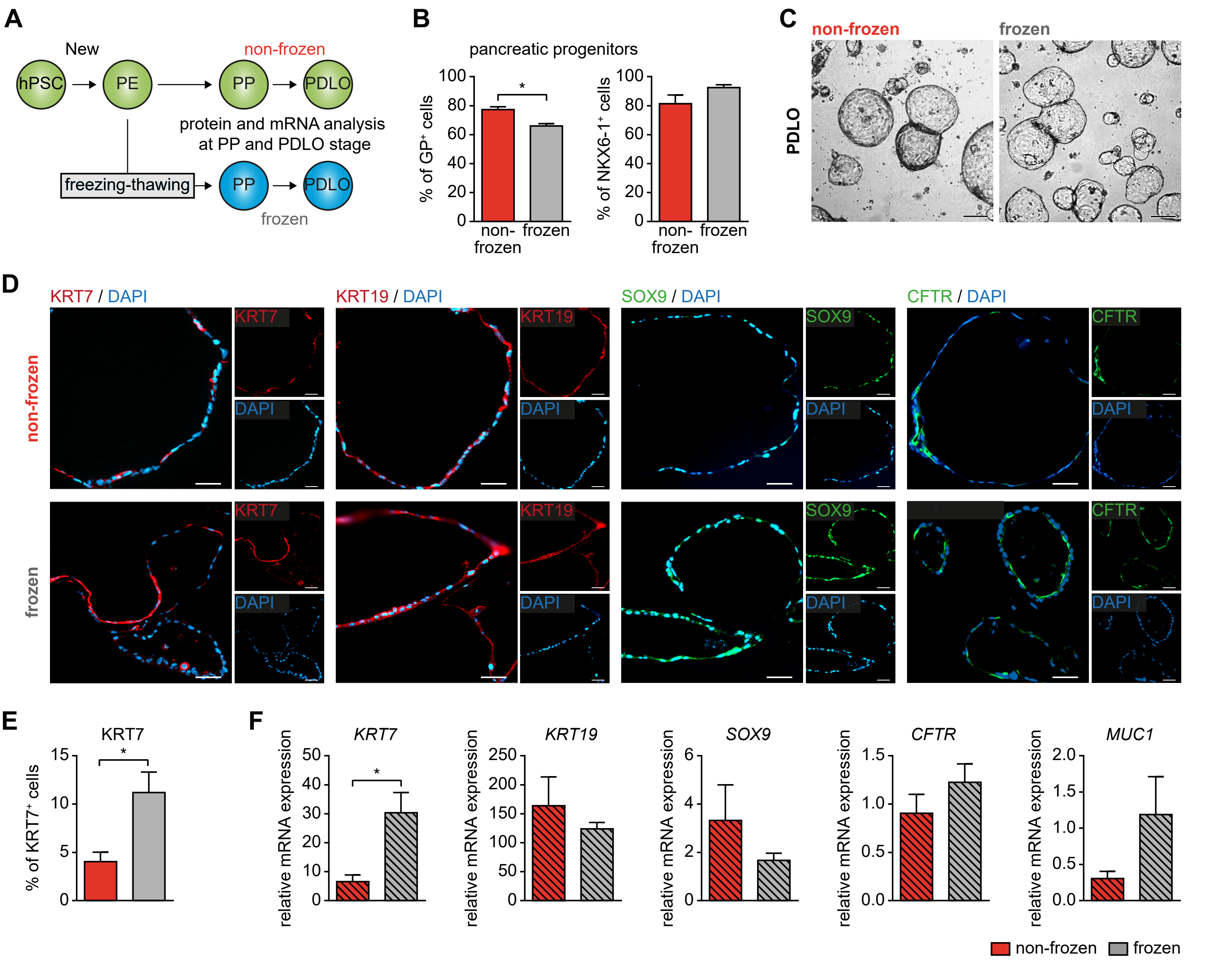
A
New
hPSC
PE
PP
PDLO
non-frozen
protein and mRNA analysis
at PP and PDLO stage
freezing-thawing
frozen
B
pancreatic progenitors
% of GP⁺ cells
% of NKX6-1⁺ cells
non-frozen
frozen
C
non-frozen
frozen
PDLO
D
KRT7 / DAPI
KRT19 / DAPI
SOX9 / DAPI
CFTR / DAPI
non-frozen
frozen
E
KRT7
% of KRT7⁺ cells
F
KRT7
KRT19
SOX9
CFTR
MUC1
relative mRNA expression
non-frozen
frozen

Figure S7. ***In vitro* ductal differentiation of new pancreatic progenitors is improved through a freeze-thaw cycle at pancreatic endoderm stage.** (**A**) Schematic illustration of PP differentiation according to the new protocol and further ductal commitment, with a freeze-thaw cycle at PE stage. (**B**) Evaluation of the progenitor markers GP2 and NKX6-1 at PP stage by flow cytometry. (**C**) Brightfield images of PDLOs derived from frozen and non-frozen PE cells. (**D**) Immunofluorescence staining of ductal organoids shows expression of the ductal markers KRT7 (red), KRT19 (red), SOX9 (green), and CFTR (green). Cells were counterstained with DAPI (blue). (**E**) Flow cytometry of KRT7 is shown. (**F**) mRNA levels of the ductal markers *KRT7*, *KRT19*, *SOX9*, *CFTR*, and *MUC1* were measured by qPCR. All gene expression data were normalized to the housekeeping gene *HMBS*. Bar graphs depict mean ± SEM, n=3. Significance was evaluated with two-tailed t-test, $P < 0.05$: *, $p < 0.01$: **, $p < 0.001$: ***. Scale bars of brightfield and immunofluorescence images represent 200 µm and 100 µm, respectively. GP2: glycoprotein 2; hPSC: human pluripotent stem cell; KRT19: keratin 19; KRT7: keratin 7; PDLO: pancreatic duct-like organoid; PE: pancreatic endoderm; PP: pancreatic progenitor

Figure S8

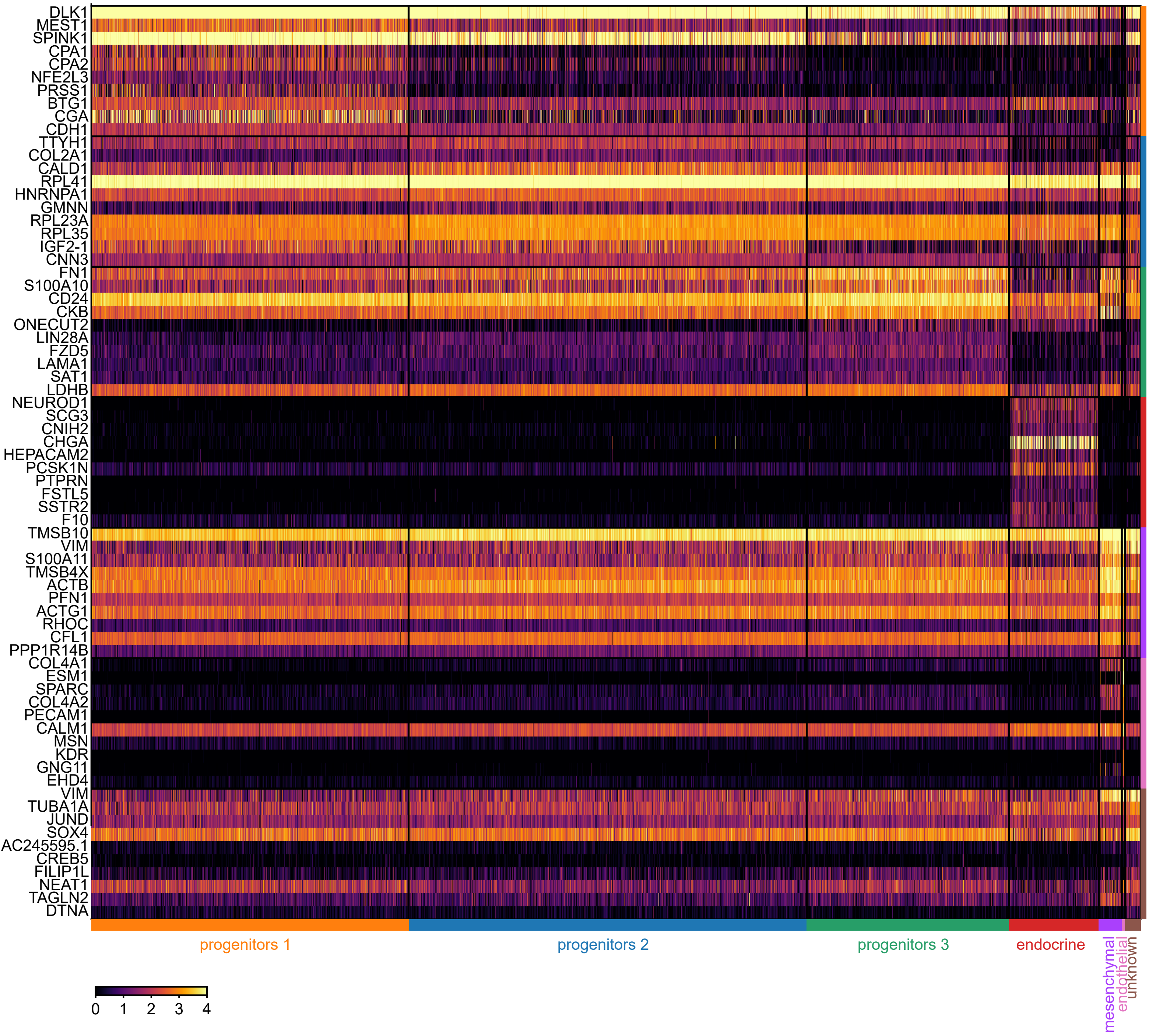
DLK1
MEST1
SPINK1
CPA1
CPA2
NFE2L3
PRSS1
BTG1
CGA
CDH1
TTYH1
COL2A1
CALD1
RPL41
HNRNPA1
GMNN
RPL23A
RPL35
IGF2-1
CNN3
FN1
S100A10
CD24
CKB
ONECUT2
LIN28A
FZD5
LAMA1
SAT1
LDHB
NEUROD1
SCG3
CNIH2
CHGA
HEPACAM2
PCSK1N
PTPRN
FSTL5
SSTR2
F10
TMSB10
VIM
S100A11
TMSB4X
ACTB
PFN1
ACTG1
RHOC
CFL1
PPP1R14B
COL4A1
ESM1
SPARC
COL4A2
PECAM1
CALM1
MSN
KDR
GNG11
EHD4
VIM
TUBA1A
JUND
SOX4
AC245595.1
CREB5
FILIP1L
NEAT1
TAGLN2
DTNA
progenitors 1
progenitors 2
progenitors 3
endocrine
mesenchymal
endothelial
unknown
0
1
2
3
4

Figure S8. Top 10 cluster-defining differentially expressed genes. Heatmap showing the top 10 differentially expressed genes specifying each cell cluster of the single-cell transcriptome of new PPs. The respective gene expression for the individual cells is shown.

Figure S9

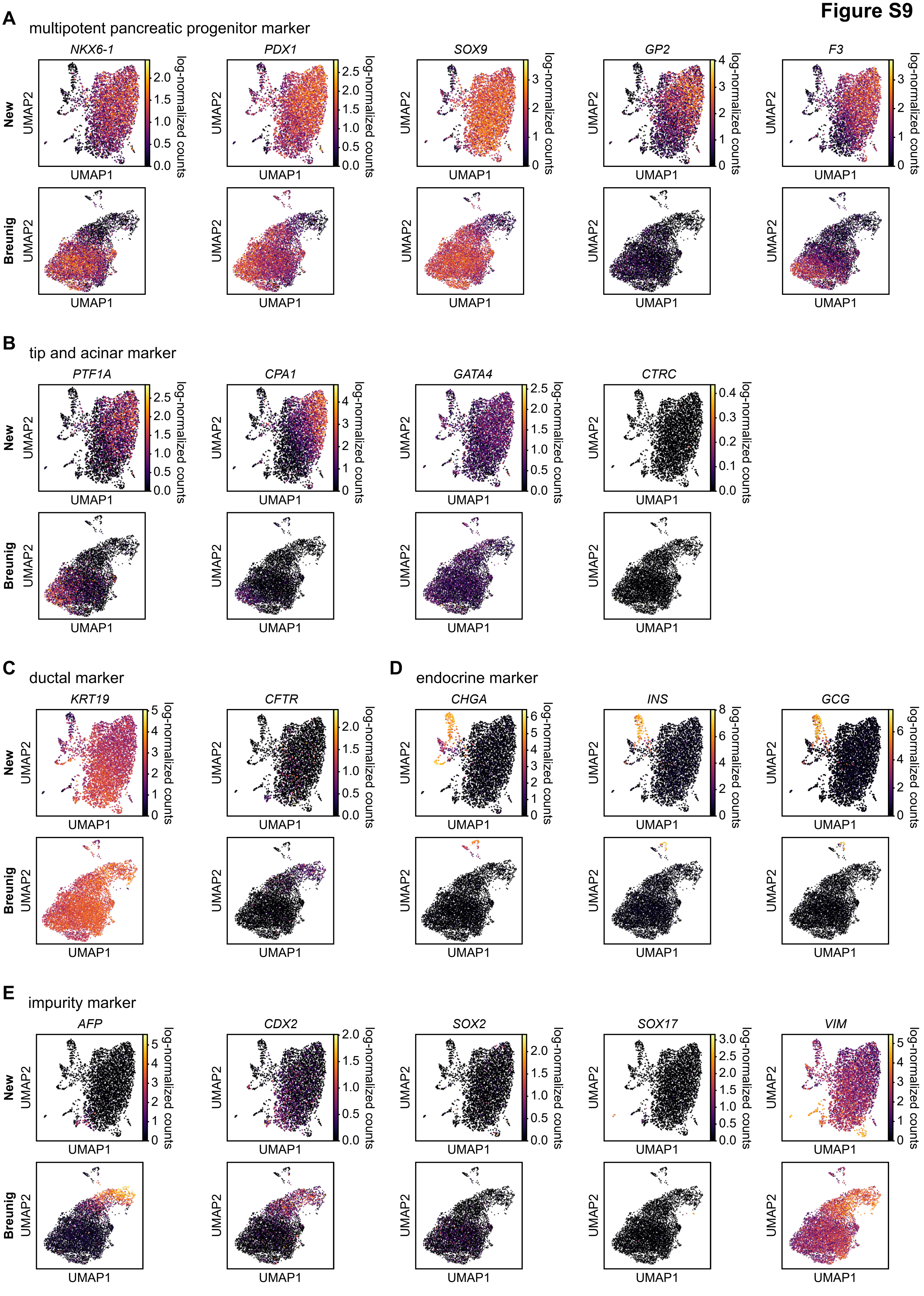
A
multipotent pancreatic progenitor marker
NKX6-1
PDX1
SOX9
GP2
F3
B
tip and acinar marker
PTF1A
CPA1
GATA4
CTRC
C
ductal marker
KRT19
CFTR
D
endocrine marker
CHGA
INS
GCG
E
impurity marker
AFP
CDX2
SOX2
SOX17
VIM
New
Breunig
UMAP1
UMAP2
log-normalized counts

Figure S9. Marker distribution within single-cell datasets of pancreatic progenitors. UMAP plots of single-cell transcriptomes within pancreatic progenitors (new and Breunig) display the expression of (**A**) multipotent pancreatic progenitor markers (*NKX6-1*, *PDX1*, *GP2*, *SOX9, F3*), (**B**) tip and acinar markers (*PTF1A*, *CPA1*, *GATA4*, *CTRC*), (**C**) ductal markers (*KRT19*, *CFTR*), (**D**) endocrine markers (*CHGA*, *INS*, *GCG*), and (**E**) non-pancreatic impurity markers (*AFP*, *CDX2*, *SOX2*, *SOX17*, *VIM*). CHGA: chromogranin A; CPA1: carboxypeptidase 1; CTRC: chymotrypsin C; GCG: glucagon; GP2: glycoprotein 2; INS: insulin; KRT19: keratin 19; VIM: vimentin

Figure S10

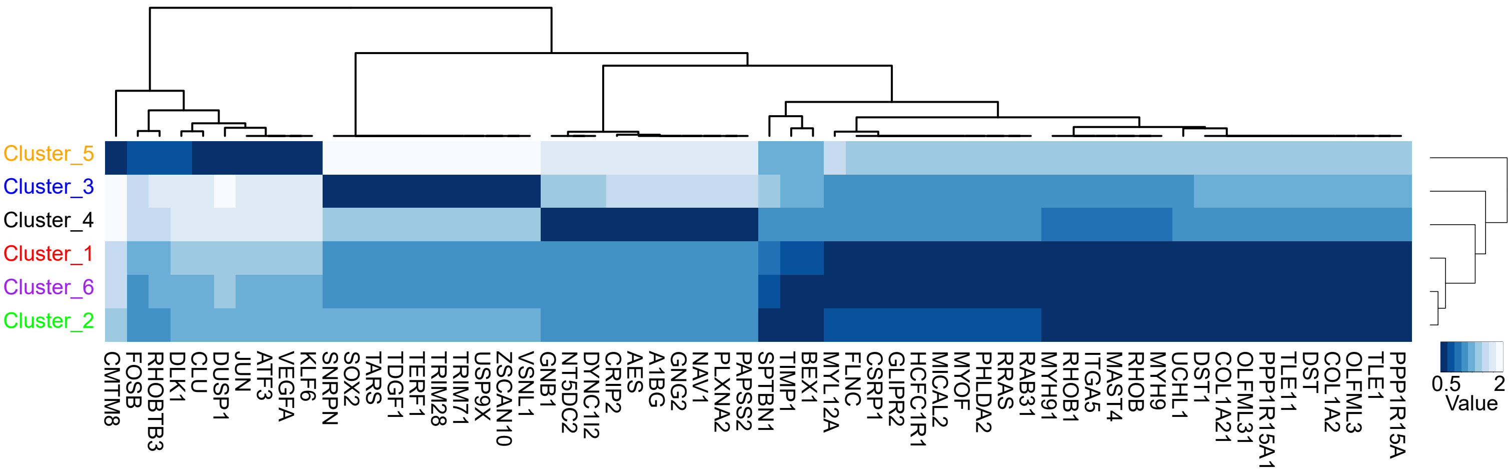

Cluster_5
Cluster_3
Cluster_4
Cluster_1
Cluster_6
Cluster_2
CMTM8
FOSB
RHOBTB3
DLK1
CLU
DUSP1
JUN
ATF3
VEGFA
KLF6
SNRPN
SOX2
TARS
TDGF1
TERF1
TRIM28
TRIM71
USP9X
ZSCAN10
VSNL1
GNB1
NT5DC2
DYNC1I2
CRIP2
AES
A1BG
GNG2
NAV1
PLXNA2
PAPSS2
SPTBN1
TIMP1
BEX1
MYL12A
FLNC
CSRP1
GLIPR2
HCFC1R1
MICAL2
MYOF
PHLDA2
RRAS
RAB31
MYH91
RHOB1
ITGA5
MAST4
RHOB
MYH9
UCHL1
DST1
COL1A21
OLFML31
PPP1R15A1
TLE11
DST
COL1A2
OLFML3
TLE1
PPP1R15A
0.5
2
Value

Figure S10. Comparison of a pancreatic progenitor dataset with a human fetal pancreas dataset leading to new cluster membership. Heatmap showing top 10 genes defining each cluster.

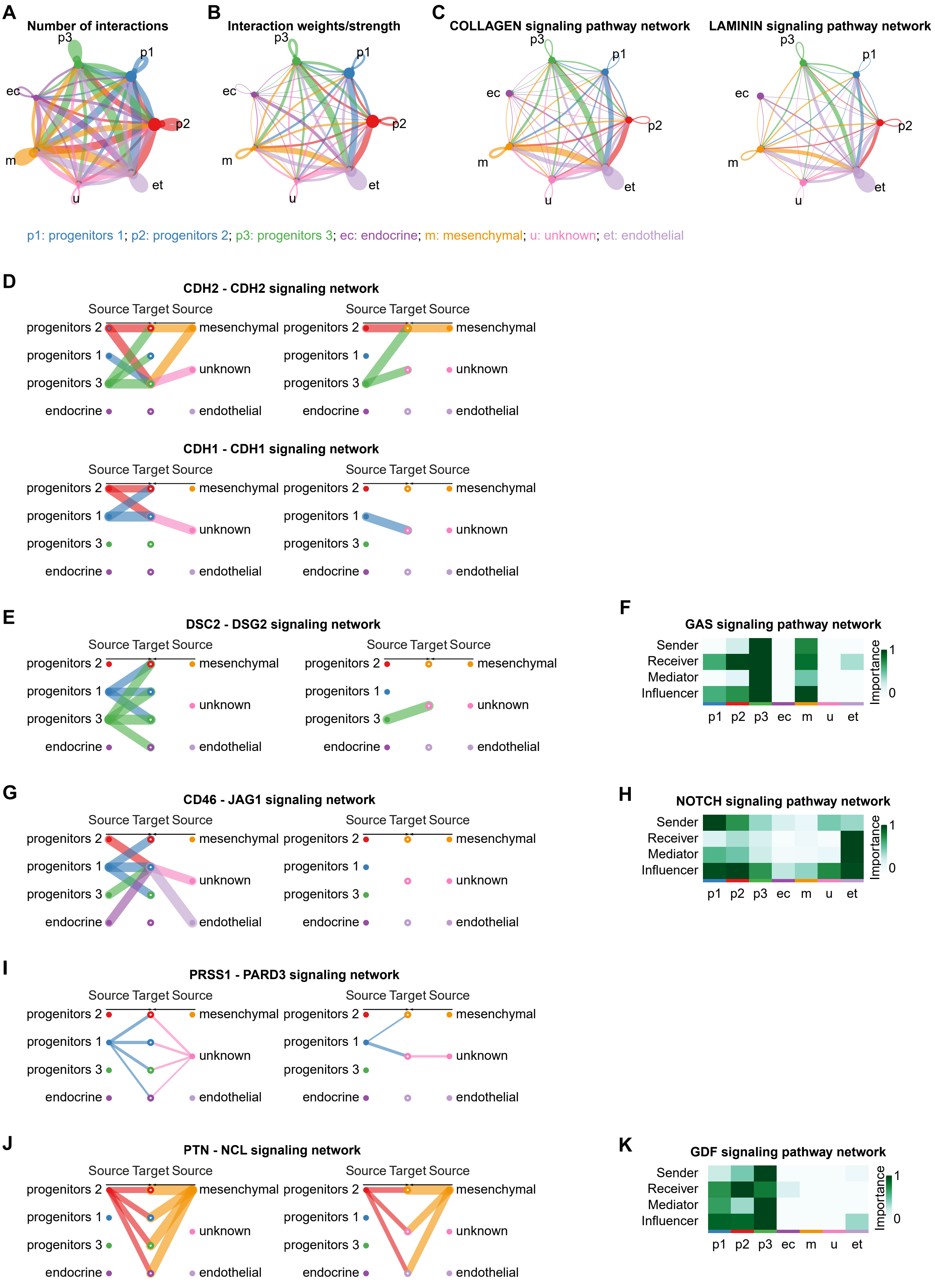
A
Number of interactions
B
Interaction weights/strength
C
COLLAGEN signaling pathway network
LAMININ signaling pathway network
p1: progenitors 1; p2: progenitors 2; p3: progenitors 3; ec: endocrine; m: mesenchymal; u: unknown; et: endothelial
D
CDH2 - CDH2 signaling network
CDH1 - CDH1 signaling network
E
DSC2 - DSG2 signaling network
F
GAS signaling pathway network
G
CD46 - JAG1 signaling network
H
NOTCH signaling pathway network
I
PRSS1 - PARD3 signaling network
J
PTN - NCL signaling network
K
GDF signaling pathway network
Source Target Source
progenitors 2
progenitors 1
progenitors 3
endocrine
mesenchymal
unknown
endothelial
Sender
Receiver
Mediator
Influencer
Importance
p1 p2 p3 ec m u et

Figure S11. Overview of cell interactions between all clusters. (**A**) Number of interactions displaying how many ligand-receptor pairs are involved in cell-cell communication. The line thickness correlates with the number of ligand-receptor pairs. The circle width resembles the number of cells involved in signaling. (**B**) Represents the intensity (probability) of cell-cell communication between the clusters. The interaction strength correlates with the thickness of the lines. (**C**) Circle plots showing Collagen and Laminin signaling pathway networks. Hierarchy plots displaying ligand-receptor signaling networks of (**D**) CDH2-CDH2 and CDH1-CDH1, (**E**) DSC2-DSG2, (**G**) CD46-JAG1, (**I**) PRSS1-PARD3, and (**J**) PTN-NCL. (**F**) GAS, (**H**) NOTCH, and (**K**) GDF signaling networks are shown as heatmap unraveling cluster-specific roles in sender, receiver, mediator or influencer. CDH1: cadherin 1; CDH2: cadherin 2; DSC2: desmocollin 2; DSG2: desmoglein 2; GAS: growth arrest specific; GDF: growth differentiation factor; NCL: nucleolin; PTN: pleiotrophin

Supplemental Table S1. Media compositions of stage 1 compound testing. Applied compounds are highlighted in green. Related to Figure 1C, D.

		Experiment number (stage 1)				
		1	2	3	4	5
Stage 1a	BE1 + 0.1% BSA	■	■	■	■	■
	1 day	■	■	■	■	■
	100 ng/mL Activin A (Peprotech)	■	■	■	■	■
	2 µM CHIR99021 (Axon MedChem)	■	■			■
	3 µM CHIR			■		
	20 ng/µL Wnt3A (PeproTech)				■	
Stage 1b	BE1 + 0.1% BSA	■	■	■	■	■
	2 days	■	■	■	■	
	3 days					■
	100 ng/mL Activin A	■	■	■	■	■
	5 ng/mL FGF2 (Novoprotein)	■		■	■	■

Supplemental Table S2. Media compositions of stage 2 compound testing. Applied compounds are highlighted in green. Related to Figure 2B.

		Experiment number (stage 2)											
		1	2	3	4	5	6	7	8	9	10	11	12
Stage 2	BE1 + 0.5% BSA	■	■	■	■	■	■	■	■	■	■	■	■
	2 days	■	■	■	■	■	■						
	3 days							■	■	■	■	■	■
	50 ng/mL KGF (PeproTech)	■		■			■		■		■	■	
	50 ng/mL FGF10 (R&D)		■		■	■		■		■			■
	0.75 µM dorsomorphin (Sigma)	■		■		■		■				■	■
	3 ng/mL Wnt3A	■			■			■			■		
	1.25 µM IWP-2 (Tocris)		■			■			■			■	
	0.25 mM vitamin C (Sigma)				■	■	■				■	■	■

Supplemental Table S3. Media compositions of stage 3 compound testing. Applied compounds are highlighted in green. Related to Figure 2C.

		Experiment number (stage 3)												
		1	2	3	4	5	6	7	8	9	10	11	12	13
**Stage 3**	BE1+ 2% BSA	■	■	■	■	■	■	■	■	■	■	■	■	■
	2 days	■	■	■	■									
	3 days					■	■	■	■	■				
	4 days										■	■	■	■
	50 ng/mL KGF	■						■		■		■		■
	50 ng/mL FGF10			■	■	■					■			
	0.25 µM SANT-1 (Sigma)	■	■	■	■	■	■	■	■	■	■	■	■	■
	1 µM retinoic acid (Sigma)		■	■			■	■		■	■			■
	2 µM retinoic acid	■			■	■			■			■	■	
	0.2 µM LDN-193189 (Sigma)		■	■		■		■		■		■	■	
	50 ng/mL murine noggin (PeproTech)	■			■		■		■		■			■
	16 mM additional glucose (in total 18.4 mM)		■		■	■	■			■		■		■
	0.5 µM PD168393 (Calbiochem)		■		■			■	■	■	■	■		
	0.5 µM TPPB (Tocris)			■	■				■	■			■	■

Supplemental Table S4. Media compositions of stage 4 compound testing. Applied compounds are highlighted in green. Related to Figure 2D.

		**Experiment number (stage 4)**														
		**1**	**2**	**3**	**4**	**5**	**6**	**7**	**8**	**9**	**10**	**11**	**12**	**13**	**14**	**15**
**Stage 4**	BE1+ 2% BSA	■	■	■	■	■	■	■	■	■	■	■	■	■	■	■
	3 days	■	■	■	■	■	■									
	4 days							■	■	■	■	■	■	■	■	■
	2 ng/mL KGF		■		■					■				■		
	50 ng/mL FGF10	■					■	■							■	
	100 ng/mL EGF (R&D)			■		■			■		■	■	■			■
	0.25 µM SANT-1	■	■			■			■		■	■		■	■	
	0.1 µM retinoic acid			■	■	■		■	■					■	■	
	0.2 µM LDN-193189		■			■	■	■	■				■	■		■
	16 mM additional glucose (in total 18.4 mM)		■	■			■		■	■		■			■	■
	10 µM SB431542 (Axon MedChem)		■		■		■		■		■		■		■	
	10 mM nicotinamide (Sigma)			■		■	■			■	■			■	■	■
	0.33 µM Indolactam V (STEMCELL Technologies)				■	■	■					■	■	■	■	■

Supplemental Table S5. Media compositions of separate PKC activator testing. Applied compounds are highlighted in green. Related to Figure 2E.

		Experiment number (PKC activator testing)				
		1	2	3	4	5
**Stage 1a**	BE1 + 0.1% BSA					
	1 day					
	100 ng/mL Activin A					
	2 µM CHIR99021					
**Stage 1b**	BE1 + 0.1% BSA					
	2 days					
	100 ng/mL Activin A					
	5 ng/mL FGF2					
**Stage 2**	BE1 + 0.5% BSA					
	3 days					
	50 ng/mL FGF10					
	3 ng/mL Wnt3A					
	0.25 mM vitaminC					
**Stage 3**	BE3 + 2% BSA					
	3 days					
	**PKC activator**		**0.1 µM ILV**	**0.33 µM ILV**	**0.5 µM ILV**	**0.5 µM TPPB**
	0.25 µM SANT-1					
	1 µM retinoic acid					
	16 mM additional glucose (in total 18.4 mM)					
	0.5 µMTPPB					
**Stage 4**	BE3 + 2% BSA					
	4 days					
	**PKC activator**	**0.33 µM ILV**	**0.1 µM ILV**	**0.33 µM ILV**	**0.5 µM ILV**	**0.5 µM TPPB**
	100 ng/mL EGF					
	10 mM nicotinamide					
	0.33 µM Indolactam V					
	16 mM additional glucose (in total 18.4 mM)					
	0.25 µM SANT-1					
	0.5 µM TPPB					

References

1. M. Breunig, J. Merkle, M. K. Melzer, S. Heller, T. Seufferlein, M. Meier, M. Hohwieler, A. Kleger. Differentiation of human pluripotent stem cells into pancreatic duct-like organoids. *STAR Protoc* **2**, 100913 (2021).
2. M. Breunig, J. Merkle, M. Wagner, M. K. Melzer, T. F. E. Barth, T. Engleitner, J. Krumm, S. Wiedenmann, C. M. Cohrs, L. Perkhofer *et al*. Modeling plasticity and dysplasia of pancreatic ductal organoids derived from human pluripotent stem cells. *Cell Stem Cell* **28**, 1105-1124 e1119 (2021).
3. M. Hohwieler, A. Illing, P. C. Hermann, T. Mayer, M. Stockmann, L. Perkhofer, T. Eiseler, J. S. Antony, M. Muller, S. Renz *et al*. Human pluripotent stem cell-derived acinar/ductal organoids generate human pancreas upon orthotopic transplantation and allow disease modelling. *Gut* **66**, 473-486 (2017).
4. N. J. Hogrebe, K. G. Maxwell, P. Augsornworawat, J. R. Millman. Generation of insulin-producing pancreatic beta cells from multiple human stem cell lines. *Nat Protoc* **16**, 4109-4143 (2021).
5. N. C. Leite, E. Sintov, T. B. Meissner, M. A. Brehm, D. L. Greiner, D. M. Harlan, D. A. Melton. Modeling Type 1 Diabetes In Vitro Using Human Pluripotent Stem Cells. *Cell Rep* **32**, 107894 (2020).
6. P. U. Mahaddalkar, K. Scheibner, S. Pfluger, Ansarullah, M. Sterr, J. Beckenbauer, M. Irmler, J. Beckers, S. Knobel, H. Lickert. Generation of pancreatic beta cells from CD177(+) anterior definitive endoderm. *Nat Biotechnol* **38**, 1061-1072 (2020).
7. N. J. Hogrebe, P. Augsornworawat, K. G. Maxwell, L. Velazco-Cruz, J. R. Millman. Targeting the cytoskeleton to direct pancreatic differentiation of human pluripotent stem cells. *Nat Biotechnol* **38**, 460-470 (2020).
